# Multimodal neurocognitive markers of frontal lobe epilepsy: Insights from ecological text processing

**DOI:** 10.1016/j.neuroimage.2021.117998

**Published:** 2021-03-28

**Authors:** Sebastian Moguilner, Agustina Birba, Daniel Fino, Roberto Isoardi, Celeste Huetagoyena, Raúl Otoya, Viviana Tirapu, Fabián Cremaschi, Lucas Sedeño, Agustín Ibáñez, Adolfo M. García

**Affiliations:** a Global Brain Health Institute, UCSF, California, US, & Trinity College Dublin, Dublin, Ireland; b Nuclear Medicine School Foundation (FUESMEN), National Commission of Atomic Energy (CNEA), Mendoza, Argentina; c University of San Andres, Buenos Aires, Argentina; d National Scientific and Technical Research Council (CONICET), Buenos Aires, Argentina; e Neuromed, Clinical Neuroscience, Mendoza, Argentina; f Neuroscience Department of the School of Medicine, National University of Cuyo, Mendoza, Argentina; g Santa Isabel de Hungría Hospital, Mendoza, Argentina; h Center for Social and Cognitive Neuroscience (CSCN), School of Psychology, Universidad Adolfo Ibáñez, Santiago, Chile; i Faculty of Education, National University of Cuyo (UNCuyo), Mendoza, Argentina; j Fundación Argentina para el Desarrollo en Salud, Mendoza, Argentina; k Universidad Católica Argentina; l Departamento de Lingüística y Literatura, Facultad de Humanidades, Universidad de Santiago de Chile, Santiago, Chile

**Keywords:** Frontal lobe epilepsy, Cognitive markers, Naturalistic discourse, Multimodal neuroimaging, machine learning

## Abstract

The pressing call to detect sensitive cognitive markers of frontal lobe epilepsy (FLE) remains poorly addressed. Standard frameworks prove nosologically unspecific (as they reveal deficits that also emerge across other epilepsy subtypes), possess low ecological validity, and are rarely supported by multimodal neuroimaging assessments. To bridge these gaps, we examined naturalistic action and non-action text comprehension, combined with structural and functional connectivity measures, in 19 FLE patients, 19 healthy controls, and 20 posterior cortex epilepsy (PCE) patients. Our analyses integrated inferential statistics and data-driven machine-learning classifiers. FLE patients were selectively and specifically impaired in action comprehension, irrespective of their neuropsychological profile. These deficits selectively and specifically correlated with (a) reduced integrity of the anterior thalamic radiation, a subcortical structure underlying motoric and action-language processing as well as epileptic seizure spread in this subtype; and (b) hypoconnectivity between the primary motor cortex and the left-parietal/supramarginal regions, two putative substrates of action-language comprehension. Moreover, machine-learning classifiers based on the above neurocognitive measures yielded 75% accuracy rates in discriminating individual FLE patients from both controls and PCE patients. Briefly, action-text assessments, combined with structural and functional connectivity measures, seem to capture ecological cognitive deficits that are specific to FLE, opening new avenues for discriminatory characterizations among epilepsy types.

## 1. Introduction

Cognitive assessments in frontal lobe epilepsy (FLE) reveal diverse deficits (Carreno and Donaire, 2008) which contribute to characterizing the pathology and its comorbidities (Braakman et al., 2013). However, most such dysfunctions (e.g., memory and fluency impairments) also occur in posterior cortex epilepsies (PCEs) (Fogarasi et al., 2003), including the more frequent temporal lobe epilepsy (Cahn-Weiner et al., 2009; Lüders et al., 1998). Moreover, cognitive studies on FLE have yielded mixed findings, with some showing deficits in frontal functions, such as memory and attention (Hernandez and Jambaque, 2003), and others reporting sparing of such domains (Risse, 2006). Furthermore, standard tests possess low ecological validity (Carreno and Donaire, 2008) and are rarely complemented with multimodal neuroimaging data. Thus, the need arises for establishing cognitive deficits that are differentially present in FLE, indicative of everyday performance, and specifically mapped to its core anatomo-functional signatures (Elger et al., 2004). A promising avenue is afforded by tasks tapping action language, a cognitive domain that hinges on cortico-subcortical motor networks (Pulvermuller, 2018, Llano, 2013, Akinina et al., 2019, Garcia et al., 2019) which are distinctively affected in FLE (Carreno and Donaire, 2008). To explore this novel view, we implemented a multimodal approach combining inferential statistics and machine learning to tap naturalistic action-language comprehension and its anatomo-functional correlates (via diffusion tensor imaging [DTI] and fMRI-derived resting-state functional connectivity [rsFC]) in FLE patients relative to both healthy controls and PCE patients.

Accounting for ≈20% of epilepsy cases (Kellinghaus and Lüders, 2004), FLE is a focal epilepsy subtype typified by brief, recurring seizures arising and spreading in the frontal lobes (Scheffer et al., 2017). Patients often exhibit damage in motor-related subcortico-cortical connections, including the anterior thalamic radiation (ATR) (Law et al., 2018); and disruptions in several rsFC networks (Cao et al., 2014), particularly involving motor network (MN) hubs (Woodward et al., 2014; Widjaja et al., 2013). Consistently, clinical manifestations include contralateral clonic movements, uni- or bilateral tonic motor activity, as well as complex automatisms (Kellinghaus and Lüders, 2004). Beyond these canonical alterations, FLE also involves deficits in numerous cognitive domains, such as attention, working memory, and verbal fluency (Carreno and Donaire, 2008). as well as consciousness states (Moguilner et al., 2017). These impairments are relevant for characterizing the disorder, but they prove inconsistent (Elger et al., 2004) and fail to differentiate it from other epilepsy types. Indeed, all such domains are also compromised in PCEs (Cahn-Weiner et al., 2009). including temporal lobe epilepsy (Cahn-Weiner et al., 2009) and less frequent subtypes such as parietal and occipital lobe epilepsies (Traianou et al., 2019). This scenario calls for new approaches to track differential neurocognitive signatures of FLE relative to PCEs at large (Elger et al., 2004).

A promising target is afforded by paradigms assessing action language, namely, verbal units denoting bodily motion (as in *Pedro took his brother’s hand and ran towards the sea*) (García and Ibàñez, 2016). This domain is critically subserved by frontal (Pulvermuller, 2018; Pulvermuller, 2013; García et al., 2019) and cortico-subcortical (Birba et al., 2017) motor circuits typically affected in FLE (Braakman et al., 2013; Woodward et al., 2014). In other neurological disorders, such as Parkinson’s disease (Abrevaya et al., 2017; Peran et al., 2003), spinocerebellar ataxia (García et al., 2016), and stroke (Akinina et al., 2019), structural and functional abnormalities along those networks entail early, selective, and primary action-language deficits (Birba et al., 2017). Moreover, action language is specifically spared in conditions compromising posterior but not anterior brain regions (Birba et al., 2017; Bak, 2003). Since FLE differs from PCEs in its marked disruption of motor systems, action-language assessments could reveal distinct neurocognitive alterations in the former.

Here we implemented a mixed hypothesis- and data-driven approach, including literature-based hypotheses and machine-learning analyses of multimodal data relevant to such predictions. Specifically, we assessed comprehension of naturalistic action texts (ATs) and neutral texts (NTs), as well as their structural (DTI) and functional (rsFC) neuroimaging signatures, in FLE patients, healthy controls, and PCE patients. Our analyses combined inferential statistics with classification algorithms (Garcia et al., 2019; Garcia et al., 2020). We raised three hypotheses. First, we predicted that, relative to controls, FLE (but not PCE) patients would exhibit selective action-language deficits. Second, given that FLE is characterized by alterations along cortico-subcortical [e.g., ATR (Law et al., 2018)] and frontal [e.g., MN (Woodward et al., 2014)] motor circuits subserving action language (Pulvermuller, 2018; Llano, 2013; Akinina et al., 2019; Abrevaya et al., 2017), we hypothesized that such deficits would correlate with reduced white matter integrity and lower rsFC across such networks –there being no comparable correlations in controls or PCE patients. Lastly, concerning machine learning analyses, we anticipated that action language outcomes would be a crucial feature for distinguishing individual FLE patients from both healthy controls and PCE patients. Briefly, our new approach aims to inform the quest for distinctive neurocognitive markers of FLE (Elger et al., 2004).

## 2. Methods

**Fig. 1.**
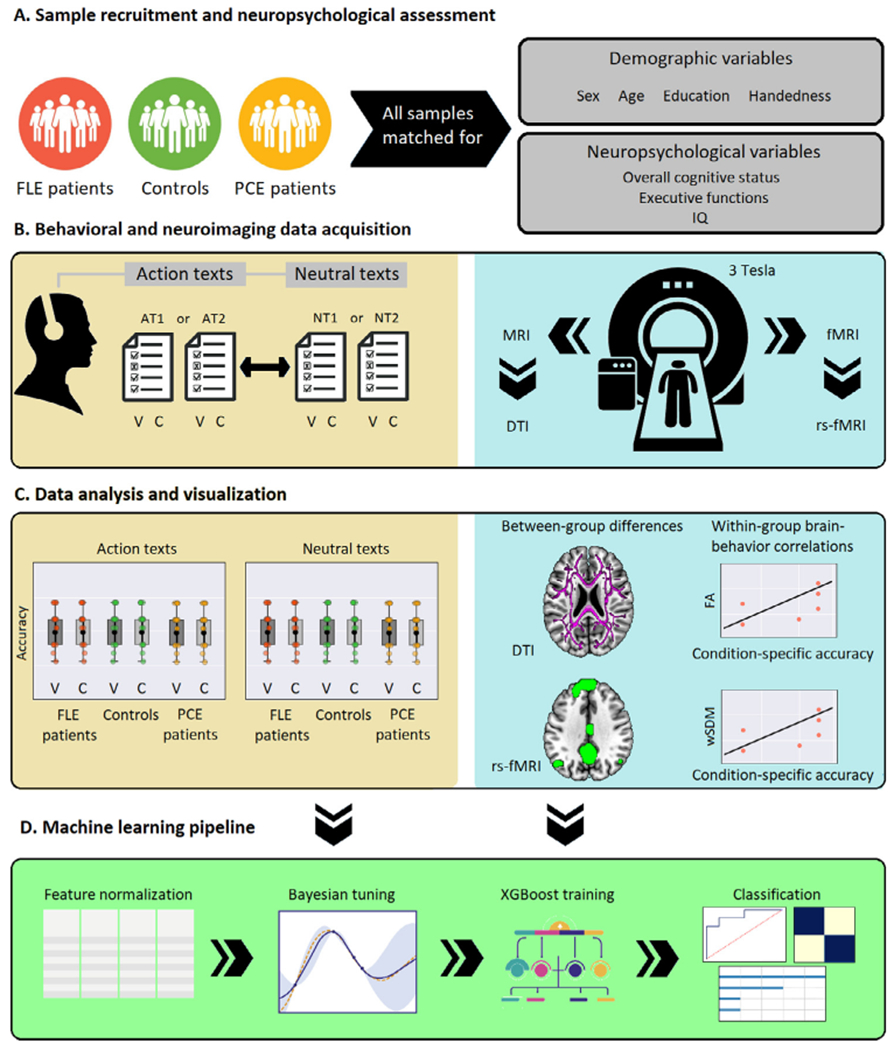
Experimental protocol. (A) Samples and neuropsychological assessment. The FLE patients, controls, and PCE patients were matched for demographical (sex, age, education, handedness) and neuropsychological (overall cognitive status, executive functions, IQ) variables. (B) Behavioral and neuroimaging data acquisition. *Left inset*. Participants listened to four recorded texts (two ATs and two NTs), presented in counterbalanced fashion. After each one, they responded to the corresponding multiple-choice questionnaire, including items on verb-related (V) and circumstantial (C) information. *Right inset*. The neuroimaging section included sequences for obtaining structural (DTI) and functional (rs-fMRI) images. (C) Data analysis and visualization. *Left inset*. Box-plot scheme for behavioral results (results for the two ATs and the two NTs were collapsed to obtain a single AT and a single NT score per subject). *Right inset*. Neuroimaging plots for DTI and rs-FC comparisons between each group pair, with their respective correlations with condition-specific (i.e., AT-V, AT-C, NT-V, NT-C) accuracy. (D) Machine learning pipeline. After feature normalization, we used a *k*-fold validation scheme for Bayesian hyper-parameter tuning to obtain trained XGBoost models, and then we tested our classification by employing the ROC curve, confusion matrices and a feature importance analysis. FLE: frontal lobe epilepsy; PCE: posterior cortex epilepsy; MoCA: Montreal Cognitive Assessment; IFS: INECO Frontal Screening battery; WASI: Weschler Abbreviated Scale of Intelligence; AT1: action text 1; AT2: action text 2; NT1: neutral text 1; NT2: neutral text 2; V: verb-related information; C: circumstantial information; DTI: diffusion tensor imaging. rs-fMRI: resting-state fMRI; rs-FC: resting-state functional connectivity; FA: fractional anisotropy; wSDM: weighted Symbolic Dependence Metric; XGBoost: eXtreme Gradient Boosting.

Our study comprised 58 participants, a sample size that reaches a power of .92 (see Supplementary Data 1). These included 19 FLE patients, showing stereotyped semiology with hypermotor seizures characterized by complex high-amplitude movements (Lüders et al., 1998); 20 PCE patients (16 with temporal lobe epilepsy, three with parietal lobe epilepsy, and one with occipital lobe epilepsy) not showing hypermotor seizures (Yu et al., 2009); and 19 healthy controls. Diagnoses were made by expert neurologists following current standards of the International League Against Epilepsy (Fisher et al., 2014). All patients had one or more confirmed clinical seizures measured by focal (i.e., not generalized) epileptic electroencephalography discharges arising and spreading through the affected lobe. Still, all of them had normal structural brain MRIs (i.e., no cortical dysplasia), as verified by a board of certified neuroradiologists. The neuroimaging and neuropsychological protocols were undertaken within a period no longer than two weeks. No patient had a history of other neurological or psychiatric disorders (evaluated via standardized neuropsychiatric testing), other disease that could cause cognitive decline, or substance abuse. The healthy controls also lacked these antecedents. The three groups were matched on age, sex, and education; handedness, determined via the Edinburgh Inventory (Oldfield, 1971); overall cognitive status, attention, and general language skills, established with the Montreal Cognitive Assessment (MoCA) and relevant subtests (Nasreddine et al., 2005); overall executive functions, working memory and inhibitory controls, assessed with the INECO Frontal Screening (IFS) battery and relevant substests (Torralva et al., 2009); and IQ, evaluated with the Weschler Abbreviated Scale of Intelligence (WASI) (Wechsler, 1999). (Fig. 1A). For details, see Table 1.

All participants provided written informed consent in accordance with The Code of Ethics of the World Medical Association (Declaration of Helsinki). The study protocol was approved by the Institutional Review Board.

### 2.1. Naturalistic text task

#### 2.1.1. Action and neutral texts

All narratives were created through a systematic protocol for establishing semantic distinctions between text sets (Birba et al., 2020a; Garcia et al., 2018). Two of the narratives were ATs, systematically focused on the characters’ bodily movements; whereas the other two were NTs, typified by low action content. Each text was based on 22 grammatical patterns that were pseudo-randomly distributed and filled with selected lexical items. These included 32 verbs strategically chosen based on semantic, syntactic, and distributional criteria to operationalize the action/non-action distinction. The number of critical items instantiating these contrasts was statistically controlled across texts. Importantly, all four texts were matched for multiple key variables, namely: character count; overall and content-word-type counts; mean content-word frequency, familiarity, syllabic length, graphemic length, and image-ability; sentence and sentence-type counts; reading difficulty; grammatical correctness, coherence, and comprehensibility; readability rating; and emotional content (Table 2). The texts communicated mostly literal meanings and contained no jargon (for full transcriptions and English translations, see supplementary data 2).

The ATs consisted in action-laden stories foregrounding their characters’ multiple bodily movements (e.g., *Johnny ran quickly to the place where the clown was jumping and dancing*). Also, the texts offered rich details about the settings where the stories took place, the objects in them, and the manner in which bodily actions were performed. Conversely, the NTs mainly described non-action events, such as the feelings, thoughts, and perceptions of their characters (e.g., *Albert was euphoric*). Also, the texts offered abundant circumstantial information depicting places, objects, and temporal features of the characters’ emotions or other internal states.

The texts were audio-recorded by a male native Spanish speaker, at a smooth pace, in .mp3 stereo format. All narrations lasted roughly 100 seconds (audio files are available online), and, were matched for voiced segments, silence segments, average fundamental frequency, and average energy (Table 2).

#### 2.1.2. Comprehension questionnaires

For each text, we designed a 20-item multiple-choice questionnaire, comprised entirely of wh- questions (Garcia et al., 2018). In each questionnaire, half the questions pointed to verb-related information, mostly based on the pattern *What did [a character] do when…?*. The other half aimed at circumstances, realized by adverbial or prepositional phrases pointing to locative, causal, temporal, or modal information signaled by the words *Where, Why, When* or *How*. All verb-related questions in the AT questionnaires referred to action verbs, and those in the NT questionnaires pointed to non-action verbs.

Questions were presented following the order of the corresponding events in the texts, with alternation between verb-related and circumstantial items. Successive questions were fully independent from each other. Each question was accompanied by one correct option, three subtly incorrect options, and an ‘I don’t remember’ option. Sequencing of the options was randomized, except for the ‘I don’t remember’ option, which always appeared last. Correct responses were given one point, while incorrect answers or the ‘I don’t remember option’ were given zero points. Therefore, each questionnaire had a maximum score of 20 points (10 for verb-related questions and 10 for questions about circumstantial information).

### 2.2. Behavioral data analysis

For all analyses, we added the scores of the two ATs, on the one hand, and those of the two NTs, of the other, yielding maximum of 20 points per condition. Following previous reports of the same task (Garcia et al., 2018), performance on each text type was separately analyzed via mixed effects models, with one between-subject factor (Group: FLE patients, PCE patients, controls) and one within-subjects factor (Information type: circumstantial, verb-related). All analyses were covaried for MoCA and IFS scores, as in (Garcia et al., 2018). Significant differences were further inspected via Tukey’s HSD tests. Alpha levels were set at *p* < 0.05. Effect sizes for main and interaction effects were calculated through partial eta squared (*η*^2^ ) tests, whereas those for pair-wise comparisons were obtained via Cohen’s *d*. All statistical analyses were performed on IBM’s SPSS Statistics (v. 23) software. The structure of the behavioral experimental session is diagrammed in Fig. 1B and C, left inset.

### 2.3. DTI methods

MRI data were acquired on a General Electric Signa PET/MR 3T scanner with a standard head coil. We obtained two types of fractional anisotropy (FA) maps. Local FA measures were used to pairwise compare WM integrity between the FLE patients, PCE patients, and controls via one-tailed two-sample *t*-tests, and global FA measures were also calculated for the correlation analyses (Section 2.6) by averaging across the obtained skeletonized tracts (for details, see Supplementary data 3). These maps were then parsed according to the Johns Hopkins ICBM DTI-based WM tract probability atlas, considering a total of 10 WM tracts (Hua et al., 2008), namely: forceps minor (Fmin), ATR, cingulate gyrus cingulum (CING), superior longitudinal fasciculus (SLF), inferior longitudinal fasciculus (ILF), corticospinal tract (CST), forceps major (Fmaj), uncinate fasciculus (UNC), hippocampal cingulum (CING-hipp), and inferior fronto-occipital fasciculus (IFOF). As recommended for this setting, which involves a large range of comparisons across voxel values, we performed a permutation-based inference while maintaining a strong control over family-wise errors (FWE) (Jenkinson et al., 2012; Nichols, 2002). This method enabled us to calculate data-driven clusters using Threshold-Free Cluster Enhancement (TFCE) (Smith, 2009), which also overcomes the need of fixing arbitrary thresholds that may bias our results.

### 2.4. fMRI methods

In the resting-state protocol, participants were asked not to think about anything in particular while remaining awake, still and with eyes closed. First, we performed a seed analysis to evaluate both linear and non-linear rsFC using the weighted Symbolic Dependence Metric (wSDM) (Moguilner et al., 2018). This measure captures local and global temporal features of the BOLD signal by weighing a copula-based dependence measure by symbolic similarity. This property enabled us to estimate dynamic nonlinear associations, a key aspect of neural connectivity that escapes the possibility of traditional metrics, like Pearson’s R –indeed, wSDM surpasses R in identifying patients with neurological disorders based on rsFC patterns (Moguilner et al., 2018). Although dependence measures, such as mutual information (MI) and weighted Symbolic Mutual Information (wSMI), tap into non-linear dependencies, their application in fMRI studies is limited because of their low temporal resolution (Kinney and Atwal, 2014). Conversely, as other dependency measures based on statistical copulas (Nelsen, 2006), wSDM uses rank statistics to circumvent this limitation. Let C be the copula function of the random variables (x , y) defined on a unit square. According to Sklar’s theorem (Sklar, 1959), there exists a unique copula C that links the joint distribution f and the marginals f_1_, f_2_:

(1) $$\text{f}\left(\text{x,y}\right)=\text{C}\left({\text{f}}_{\text{1}}\left(\text{x}\right){\text{,f}}_{\text{2}}\left(\text{y}\right)\right)$$

Using the result that the variables x , y are independent if and only if the copula C equals the product copula ∏ defined as the product of their marginal distribution functions (Nelsen, 2006), the independence of the variables can be measured by a normalized L^P^ distance of C and ∏:

(2) $${\left(h_{p}\int \int_{{\left[0,1\right]}^{2}}|\text{C}\left({\text{u}}^{\text{1}}{\text{u}}^{\text{2}}\right)-\prod \left({\text{u}}^{\text{1}}{\text{u}}^{\text{2}}\right)|{\text{du}}^{\text{1}}{\text{du}}^{\text{2}}\right)}^{\frac{1}{\text{p}}},$$

where 1 ≤ p ≤ ∞ and h_p_ is a normalization constant.

For p = 2, we have Hoeffding’s phi-square (I*ϕ*^2^ ) (Hoeffding, 1940),

(3) $$\text{I}\prod^{2}=90\int \int_{{\left[0,1\right]}^{2}}|\text{C}\left({\text{u}}^{\text{1}}{\text{,u}}^{\text{2}}\right)-\prod \left({\text{u}}^{\text{1}}{\text{,u}}^{\text{2}}\right)|{\text{du}}^{\text{1}}{\text{du}}^{\text{2}}$$

whose empirical estimation can be analytically computed (Gaißer et al., 2010). The coefficients of the wSDM formula (Moguilner et al., 2018) were obtained through the Information Theoretical Estimators Toolbox (Szabo, 2014). Finally, to account for local variations in the time-series, we represented the increase and decrease of the signal by symbols. That allowed us to perform comparisons of sequences of symbols, enabling a dynamical analysis of the dependence between regions. To this end, we defined a symbolic weight sw, which is function of the similarity of $\hat{\text{X}}$, $\hat{\text{Y}}$ (i.e., the symbolic transformation of the x , y timeseries), and multiplying the copula-based measure I(x , y) we obtain the formula for the wSDM:

(4) $$\text{wSDM}=\text{sw}\left(\hat{\text{X}},\hat{\text{Y}}\right).\text{I}\left(\text{x},\text{y}\right)$$

The symbolic weights, which range from 0 (i.e., minimal similarity) to 1 (i.e., maximal similarity), were calculated using the Hamming distance (Lesk, 2002) between the obtained symbolic strings.

Our analysis targeted three different networks. First, we considered a critical motor network (MN), implicated in action planning, execution, observation, as well as in embodied semantic processes during action imagery and action-language (Hauk et al., 2004) tasks. Second, as a domain-specific (semantic) control, we examined a multimodal semantic network (SemN), associated with processing of integrative, modality-neutral concepts (Lambon Ralph et al., 2017). Finally, as a functionally unspecific control, we assessed the visual network (VN), which plays no distinctive roles in semantic processing (for details, see Supplementary data 4).

### 2.5. Correlation analyses

For each group separately, we performed linear correlations between scores from the four conditions of the naturalistic text task (i.e., verb-related information in the ATs, circumstantial information in the ATs, verb-related information in the NTs, circumstantial information in the NTs) and measures of (a) structural and (b) functional brain networks. The former were based on the averaged FA in the tracts parcellated with the JHU atlas (10 structures). The latter considered the averaged fMRI functional connectivity maps of the seeds each rsFC networks (i.e., the MN, the SemN, and the VN). Given that the data was normally distributed for both the neuroimaging and naturalistic text task outcomes (FA data: Shapiro–Wilk test, *p* = 0.12; rsFC data: Shapiro–Wilk test, *p* = 0.21; AT data: Shapiro–Wilk test, *p* = 0.11; NT data: Shapiro—Wilk test, *p* = 0.13), correlations were examined via Pearson’s correlation coefficient, at a threshold of *p* < .05, corrected for multiple comparisons among correlations via FDR (Cai and Liu, 2016). This method is adequate when multiple associations are being evaluated between complex neuroimaging measures and a behavioral task with a restricted range of possible values, as it controls the expected proportion falsely rejected hypotheses better than more restrictive procedures (Genovese et al., 2002). The structure of the neuroimaging experimental session is diagrammed in Fig. 1B and C, right inset.

### 2.6. Machine learning analysis

Following machine-learning analysis guidelines (Dobbin and Simon, 2011), we split the datasets in a ratio of 80% for training, and 20% for testing, using random division, to test for generalizability without employing the testing dataset during the validation phase for out-of-folds predictions (for details, see Supplementary data 5). The 80/20 split is the gold-standard for obtaining robust cross-validation results across fields (Poldrack et al., 2017), including neuroimaging research (e.g., (Lanka et al., 2020)), in general, and neurolinguistic studies (e.g., (Soto et al., 2020)), in particular. We trained the model with all the set of normalized features (i.e., verb-related and circumstantial information outcomes in each text type, FA results for the 10 JHU atlas tracts, and results for each of the seeds in each of the rsFC networks). For the training phase in all our analyses, following best practices, we employed a *k*-fold cross-validation for hyper-parameter tuning (Poldrack et al., 2019). First, we ran a classifier to discriminate between FLE patients from controls. Then, to test the specificity of potential results from that analysis, we examined the classification accuracy between PCE patients and controls, and then between FLE and PCE patients. To establish which features were the most relevant for each classification scheme, we employed the feature importance analysis technique, built-in in our machine learning algorithm (Chen and Xgboost, 2016). We used a GBM classifier library called eXtreme Gradient Boosting (XG-Boost) (Chen, 2016), because of its high accuracy and robustness relative to other algorithms, tuning its hyper-parameters by Bayesian Optimization (Zeng and Luo, 2017; Feurer, 2019). GBMs are based on the gradient boosting technique, in which ensembles of decision trees iteratively attempt to correct the classification errors of their predecessors by minimizing a loss function (i.e., a function representing the difference between the estimated and true values) while pointing in the negative gradient direction (Mason LB and Bartlett, 1999). The XG-Boost classifier provides parallel computation tree boosting, enabling fast and accurate predictions which have proven successful in several fields (Behravan et al., 2018; Zheng et al., 2017; Torlay et al., 2017); and also regularized boosting, helping to reduce overfitting and thus providing more generalizable results.^63^, ^64^ Following guidelines for reporting machine learning results (Uddin et al., 2019), classification accuracy values were accompanied by (i) calculations of the area under the curve (AUC) of the receiver operating characteristic (ROC) curve, and (ii) confusion matrices capturing the sensitivity and specificity of each classification. The machine learning pipeline is diagrammed in Fig. 1D.

To further test the robustness of naturalistic language measures relative to standard cognitive tasks, we performed an additional machine learning analysis (employing the same pipeline as in the main analysis), incorporating outcomes from five domain-general measures (subtests of visuospatial, attentional, language, abstraction, and delayed recall from the MoCA) and five executive measures (subtests of motor programming, conflicting instructions, inhibitory control, proverb interpretation, and working memory from the IFS).

### 2.7. Data availability

All experimental data, as well as the scripts used for their collection and analysis, are fully available online (Moguilner, 2020).

## 3. Results

### 3.1. Behavioral results

**Fig. 2.**
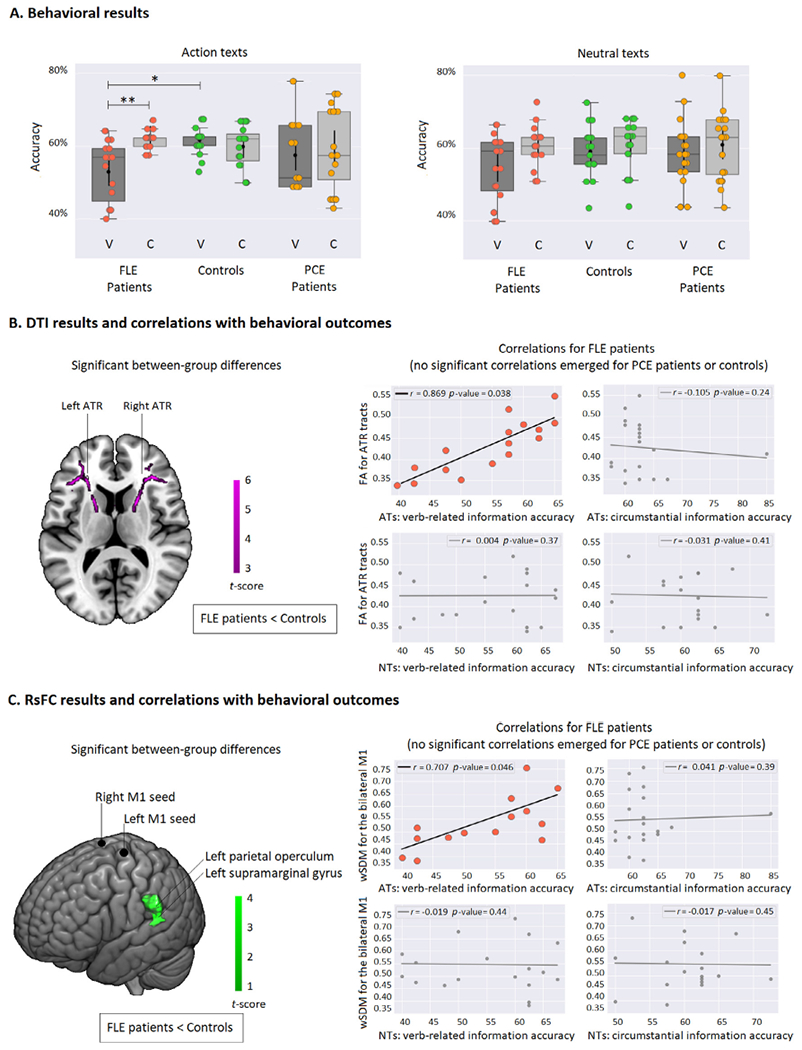
Behavioral, neuroimaging, and correlation results. (A) Behavioral results. The AT yielded a significant deficit for verbs (action verbs) in FLE patients, relative to both circumstances in the same group and verbs in controls (no other pairwise contrast proved significant for the AT). The NT revealed non-significant differences among groups or within any individual group. All results were covaried for MoCA and IFS scores. (B) DTI results and correlation with behavioral outcomes. *Left inset*. Significant between-group differences for the FLE patients < Controls contrast in FA measures, revealing reduced white matter tract integrity in the ATR. No differences were observed between PCE patients and any of the other two groups. *Right inset*. In FLE patients, FA of the ATR tracts selectively correlated with accuracy for verbs in the ATs. No significant correlations emerged for any other tract in FLE patients nor for any tract at all in the other two groups. (C) Rs-FC results and correlation with behavioral outcomes. *Left inset*. Significant between-group differences for the FLE patients < Controls contrast in the wSDM functional connectivity map, showing reduced connectivity between the bilateral M1 region and the left parietal operculum and left supramarginal gyrus. *Right inset*. In FLE patients, significant M1-posterior hypo-connectivity selectively correlated with accuracy for verbs in the ATs. No significant correlations emerged for any other rs-FC seed in FLE patients nor for any seed at all in the other two groups. Asterisks (*) indicate significant differences. FLE: frontal lobe epilepsy; PCE: posterior cortex epilepsy; V: verb-related information; C: circumstantial information; DTI: diffusion tensor imaging; rs-fMRI: resting-state fMRI; re-FC: resting-state functional connectivity; FA: fractional anisotropy; wSDM: weighted Symbolic Dependence Metric; ATR: anterior thalamic radiations; ATs: action texts; NTs: neutral texts.

The AT yielded non-significant main effects of group [*F*(2,110) = 1.39, *p* = 0.25, *η*^2^ = 0.02] and a significant main effect of information type [*F*(1,110) = 3.01, *p* = 0.02, *η*^2^ = 0.1], with lower outcomes for verbs than circumstances. This pattern survived covariation with MoCA scores [*F*(2,110) = 8.19, *p* = 0.01, *η*^2^ = 0.28)] but not with IFS scores [*F*(2,110) = 1.14, *p* = 0.70, *η*^2^ = 0.09)]. More crucially, a significant interaction emerged between group and information type, which was preserved after covariation with MoCA and IFS scores [*F*(2,110) = 8.14, *p* = 0.01, *η*^2^ = 0.26)]. A post-hoc analysis, via Tukey’s HSD test (MSE = 65.881, *df* = 104.63), revealed a significant selective effect in the FLE group, with verb-related questions yielding lower outcomes compared to circumstantial questions in the same group (*p* = 0.01, *d* = 0.95) and to verb-related question in the control group (*p* = 0.03, *d* = 0.85) (Fig. 2A). Every other pair-wise comparison within and across FLE patients, controls, and PCE patients yielded non-significant differences (all *p*-values > 0.10). For details, see Supplementary data 6.

As regards the NT, results revealed non-significant effects of group [*F*(2,110) = 0.309, *p* = 0.73, *η*^2^ = 0.006] and information type [*F*(1,110) = 3.56, *p* = 0.5, *η*^2^ = 0.032], as well as a non-significant interaction between both factors [*F*(1,110) = 0.387, *p* = 0.68, *η*^2^ = 0.007]. For details, see Supplementary data 7.

### 3.2. DTI results

Local FA measurements revealed significantly lower WM integrity (*p* < 0.05, FWE corrected) for FLE patients than controls in bilateral segments corresponding to the ATR tract (Fig. 2B, left inlet). No tract exhibited higher FA for FLE patients than controls. Moreover, no other local FA pairwise comparison between subject groups showed significant differences in any tract. For details, see Supplementary data 8.

Global FA measures, averaged within the 10 JHU atlas tracts, showed significantly lower WM integrity for FLE patients than controls [*t*(18) = 2.45, FDR-corrected *p* = 0.03, *d* = 0.83] in the bilateral ATR tract. No other tract showed significant differences between FLE patients and controls in any direction. Also, no other global FA pairwise comparison between subject groups showed significant differences in any tract. For details, see Supplementary data 9.

### 3.3. fMRI results

Relative to controls, FLE patients exhibited MN hypoconnectivity, indexed by significantly lower (FDR-corrected *p* < 0.05) rsFC between the bilateral M1 seeds and a cluster over the left parietal operculum and supramarginal gyrus (Fig. 2C, left inlet). The cluster’s peak *t*-score [*t*(18) = 3.58, *p* = 0.001, *d* = 0.87] was located in the following MNI coordinates: −50, −42, 24. No other seed yielded significant rsFC differences in any of the remaining pairwise comparisons between FLE patients, controls and PCE patients (all *p*-values > 0.13). For details, see Supplementary data 10.

### 3.4. Correlation analysis results

In FLE patients, a strong positive correlation (*r* = 0.869, FDR-corrected *p* = 0.03) emerged between FA in the ATR tract and verb-related AT accuracy scores (i.e., action comprehension) (Fig. 2B, right inlet). Every other correlation between FA and performance proved nonsignificant across groups, tracts, and conditions (all *p*-values > 0.21). For details, see Supplementary data 11.

In the FLE group, we found a strong positive correlation (*r* = 0.707, FDR-corrected *p* = 0.04) between averaged rsFC from the bilateral MN seed and verb-related AT accuracy scores (i.e., action comprehension) (Fig. 2C, right inlet). Every other correlation between wSDM and performance proved non-significant across groups, seeds, and conditions (all *p*-values > 0.09). For details, see Supplementary data 12.

### 3.5. Machine learning results

**Fig. 3.**
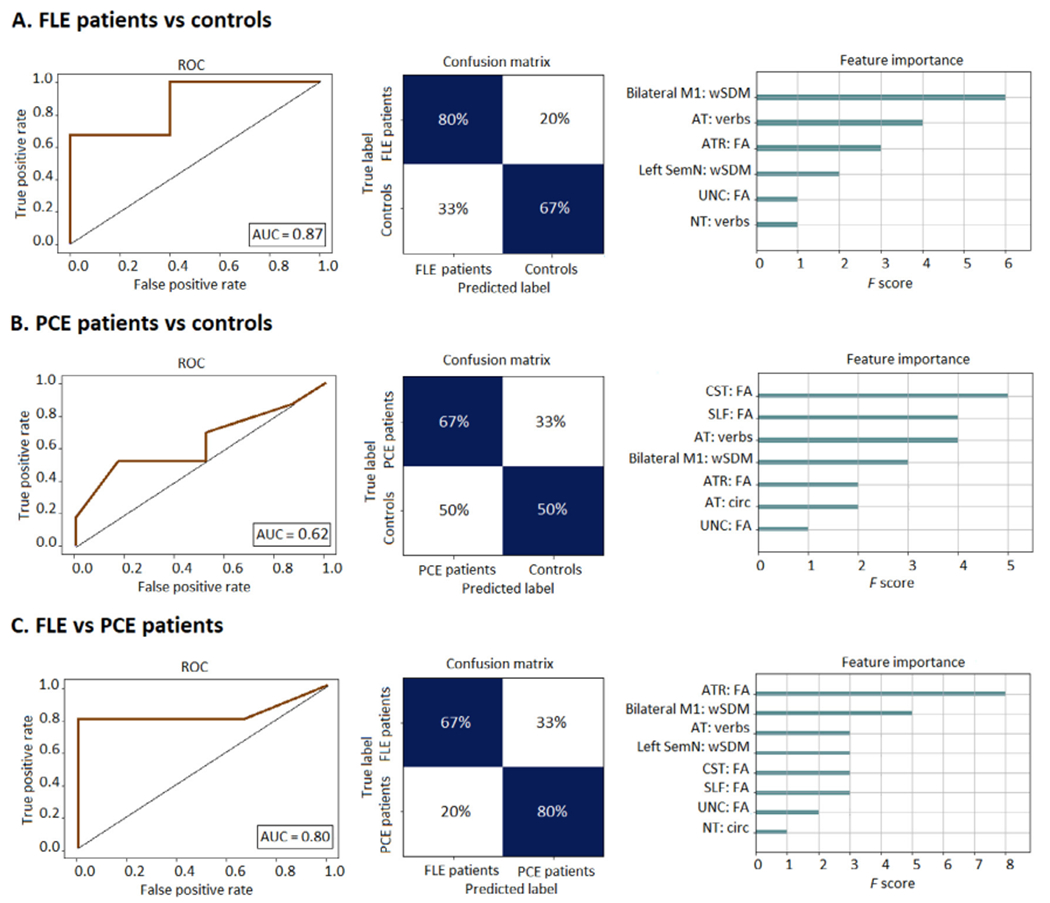
Machine learning results. (A) FLE patients vs controls. ROC curve indicating specificity (true positive rate) and sensitivity (false positive rate), while calculating the area under the curve. Confusion matrix for true label and predicted label accuracy details. Feature importance plot of the most relevant features for the classification. Results show a 75% accuracy rate, an AUC of 0.87, a sensitivity of 80%, and a specificity of 66.67%, with the bilateral M1: wSDM value as the top feature, followed by AT verbs. (B) PCE patients vs controls. ROC curve indicating specificity (true positive rate) and sensitivity (false positive rate), while calculating the area under the curve. Confusion matrix for true label and predicted label accuracy details. Feature importance plot of the most relevant features for the classification. Results yielded a near-chance accuracy rate (58.33%), with an AUC of 0.62, with a sensitivity of 66.67% and a specificity of 50% specificity (C) PCE vs FLE patients. ROC curve indicating specificity (true positive rate) and sensitivity (false positive rate), while calculating the area under the curve. Confusion matrix for true label and predicted label accuracy details. Feature importance plot of the most relevant features for the classification. Results yielded a 75% accuracy rate, an AUC of 0.80, a sensitivity of 66.67%, and a specificity of 80%, with the ATR FA value as the top feature, followed by bilateral M1 wSDM and then by verb-related AT outcomes. ROC: Receiver operating characteristic, AUC: Area under the curve, FLE: Frontal lobe epilepsy, PCE: Posterior cortex epilepsy, ATR: anterior thalamic radiations, SLF: superior longitudinal fasciculus, UNC: uncinate fasciculus, M1: Primary motor cortex, wSDM: weighted Symbolic Dependence Metric.

The machine learning classification between FLE patients and healthy control groups, based on an XGBoost algorithm that included all behavioral conditions, with all WM tracts, and all rsFC network features, achieved a 75% accuracy rate. The classificatory relevance was highest for the bilateral M1 wSDM feature, followed by verb-related AT scores and the ATR FA, and then by other less relevant features. The ROC curve showed an AUC of 0.87, with 80% sensitivity and 66.67% specificity shown in the confusion matrix (see Fig. 3A for details).

The classification between PCE patients and healthy control groups, based on an XGBoost algorithm that included all behavioral conditions, with all WM tracts, and all rsFC networks features, achieved a near chance (58.33%) accuracy rate. The classificatory relevance was highest for the CST tracts, followed by the SLF and the verb related AT, and then by other less relevant features. The ROC curve showed an AUC of 0.62, with a sensitivity of 66.67% and a specificity of 50% (see Fig. 3B for details).

The classification between FLE and PCE patients based on an XG-Boost algorithm that included all behavioral conditions, with all WM tracts, and all rsFC networks features, achieved a 75% accuracy rate. The classificatory relevance was highest for ATR FA, followed by the bilateral M1 wSDM value and verb-related AT scores, and then by other less relevant features. The ROC curve showed an AUC of 0.80, with 66.67% sensitivity and 80% specificity (see Fig. 3C for details).

Finally, to assess the relevance of considering the naturalistic task features, we executed the same classification analyses but only considering the FA of the WM tracts and rsFC networks. Importantly, the classification results reported above were markedly higher than those obtained upon exclusion of the linguistic variables, indicating that action-verb comprehension is a substantial contributor to the differentiation of FLE patients from both controls and PCE patients (see details in Supplementary data 13). An additional machine learning analysis including diverse cognitive and executive scores corroborated that action-language processing and its neural correlates remained at the top of the feature importance rankings distinguishing FLE patients from both controls and PCE patients. Conversely, such coarse-grained cognitive variables had negligible contribution to classification accuracy, highlighting the relevance of our naturalistic tasks (see Supplementary data 14).

## 4. Discussion

Through a combination of multimodal (behavioral, tractographic, and rsFC) measures, inferential statistics, and data-driven machine learning, our study revealed differential and ecological neurocognitive markers of FLE. Unlike PCE patients, those with FLE had selective action discourse deficits specifically associated with structural and functional alterations along motor-related networks. Moreover, that selective deficit had major weight in discriminating individual FLE patients relative to controls and, more importantly, PCE patients. Below we discuss these findings in detail.

FLE patients exhibited selective verb-related deficits in the ATs and no deficits in either NT category. This pattern, previously observed in Parkinson’s disease (Garcia et al., 2018), points to highly focal impairments in action comprehension, as opposed to language or even verb-related information in general. Indeed, selective action-semantic difficulties are systematic across disorders presenting frontal motor-network damage, including Parkinson’s, Huntington’s, and motor-neuron disease as well as amyotrophic lateral sclerosis (Garcia et al., 2018). Crucially, this deficit was *exclusive* to the FLE group. PCE patients had preserved outcomes in all text categories, corroborating previous evidence of spared action semantics following posterior cortical damage (Bak, 2003).

Of note, in the AT, verb scores were overall lower than those of circumstantial questions, replicating previous results from the same task in Parkinson’s disease patients (García et al., 2018) and corroborating that verbs may involve greater processing demands than other word categories (Vigliocco et al., 2011). Interestingly, this effect remained after covariation with MoCA scores, but not with IFS scores, suggesting that processing of this category may be more related to executive function rather than overall cognitive status −although more research would be needed to directly test this conjecture.

Importantly, however, patients exhibited normal MoCA and IFS scores, all the key interaction effect survived covariation for both measures, and key subtests from these instruments exhibited negligible contribution in complementary classification analyses, highlighting the discriminatory value of our naturalistic measures (see Supplementary material 14). Hence, as reported in other populations (Garcia et al., 2018), the selective and differential action-comprehension deficits observed in FLE patient cannot be attributed to domain-general cognitive dysfunction. This underscores the relevance of our text-based results, showing that action-language tasks can capture significant and selective deficits even when classical measures fail to do so.

Such deficits were distinctively correlated with reduced white matter integrity along the ATR, a tract that was preserved in PCE and which is often compromised in FLE (Law et al., 2018). ATR alterations underlie motor-function decay in healthy adults (Philp et al., 2014) and neurological conditions (Isaacs et al., 2019) typified by action-semantic deficits (Birba et al., 2017). Indeed, this and other subcortical motor structures are directly implicated in action-language processing (Llano, 2013; Akinina et al., 2019), and their anatomical disruption correlates with the recruitment of non-canonical cortical motor pathways for action-verb access (Abrevaya et al., 2017). Accordingly, the differential impairment observed in FLE was also related to putative structural networks distinctively affected in this epilepsy subtype (Lin et al., 2020).

Action comprehension deficits in FLE were also specifically correlated with hypoconnectivity between M1 and left parietal/supramarginal regions. Whereas action-verb processing critically hinges on cortical motor circuits (Pulvermuller, 2018; Pulvermuller, 2013; Birba et al., 2020a). It has also been systematically related to secondary contributions of posterior areas that subserve multimodal semantics (Garcia et al., 2019), including parietal and supramarginal hubs (Pulvermuller, 2018). In fact, selective action-verb impairments in other neurological conditions entail aberrant functional connectivity between motor and posterior regions underpinning general semantic processes (Abrevaya et al., 2017). Importantly, no other correlation with rsFC patterns emerged in either FLE or PCE patients, further highlighting the specificity of the former’s neurocognitive disruption.

Moreover, action comprehension deficits were highly relevant to identify *individual* FLE patients. Machine learning results yielded 75% accuracy in discriminating these subjects from both controls and, more crucially, PCE patients. In these settings, action comprehension showed high feature importance, even surpassing other structural and functional network markers. Indeed, the only two features with greater weight corresponded to tractographic and rsFC substrates specifically associated to action verbs —namely, the ATR (Llano, 2013; Akinina et al., 2019) and functional motor networks (Abrevaya et al., 2017; Birba et al., 2020a). Furthermore, removal of action comprehension outcomes from the classifiers substantially reduced patient identification (Supplementary data 13), which emphasizes their discriminatory relevance. In the same vein, comprehension of actions in naturalistic stories was shown to outper-form validated executive function tests in identifying patients with frontobasal atrophy, yielding over 80% accuracy (Garcia et al., 2018). These findings, together with the near-chance classification outcomes found for PCE patients vis-à-vis controls, suggest that action comprehension may contribute to the differentiation between FLE and PCE even on a subject-by-subject basis.

An additional highlight, in this sense, is that our task was based on highly ecological texts. As argued elsewhere (Carreno and Donaire, 2008), cognitive dysfunctions in frontal disorders are better tracked under naturalistic conditions. Yet, traditional neuropsychological tests (tapping such domains as working memory, visuo-constructional skills, calculation, or visual memory) (Kurzbuch et al., 2013) rely on decontextualized and randomly sequenced materials, potentially leading to false negatives (Carreno and Donaire, 2008; Elger et al., 2004). Our naturalistic text framework partly overcomes these shortcomings by capturing key aspects of daily language processing with context-rich narratives characterized by cohesion, coherence, and unfolding semantic relations (Birba et al., 2020a; Garcia et al., 2018; Trevisan and García, 2019; Franceschini et al., 2017). Accordingly, our approach also meets recent demands for more ecological assessments of language (Verga and Kotz, 2018), in general, and FLE (Carreno and Donaire, 2008), in particular. More specifically, this approach informs recent calls for studies that inform neurocognitive models of naturalistic language processing (Verga and Kotz, 2018), including embodied domains (Birba et al., 2020b).

Taken together, our results address recent calls for finding specific cognitive markers of FLE (Elger et al., 2004). Classical cognitive tests in epilepsy show that FLE patients are often impaired in attention, memory, verbal fluency, and language processing (Carreno and Donaire, 2008). However, each of these domains is also affected in PCE (Cahn-Weiner et al., 2009), which undermines their discriminatory value as specific cognitive markers of FLE. Moreover, coarse-grained cognitive profiles in epilepsy are heterogeneous, with diverse pathophysiological mechanisms influencing their manifestation across patients (Elger et al., 2004) Our embodied framework, focused on action semantics, seems to circumvent this limitation by revealing deficits that are exclusive to FLE, correlated with core anatomo-functional alterations of this disorder, (Pulvermuller, 2018, Llano, 2013, Akinina et al., 2019), (Pulvermuller, 2013 Sep) and robust for discriminating individual FLE and PCE patients when combined with structural and functional connectivity measures. Thus, unlike standard cognitive tasks, paradigms tapping action semantics and relevant multimodal neuroimaging markers could complement differential diagnosis tools and even support estimations of the course of pathology across patients. (Birba et al., 2017 Aug 02)

### 4.1. Limitations and avenues for future studies

Our work is not without limitations. First, although our sample size was similar to or larger than those of previous reports (Cahn-Weiner et al., 2009; Lambon Ralph et al., 2012) and it conferred high statistical power (see Section 2.1), it would be desirable to replicate this experiment with larger *Ns.* Second, whereas our neuropsychological protocol including several tasks and subtasks tapping diverse cognitive domains, future renditions should incorporate additional classical tests for comparison across epilepsy subtypes. Moreover, further work would be necessary to ascertain the extent to which this new paradigm can reveal distinguishing neurocognitive traits of FLE in clinical settings. Third, behavioral outcomes were not measured whilst the subject was in the scanner. Even though this was a strategic choice to prevent poor audibility within the scanner, future adaptations should explore *in vivo* signatures of naturalistic text processing, as recently done in electroencephalographic research (Birba et al., 2020a). Fourth, we lacked detailed information about the patients’ medication status. Given that neurotransmitter bioavailability may modulate action language processing (Herrera et al., 2012), new studies should factor this variable in. Fifth, beyond our focus on FLE, our framework lays the groundwork for new embodied designs seeking specific markers of other epilepsy types. Finally, our study collapsed all non-frontal subtypes within a single PCE group (Fogarasi et al., 2003; Yu et al., 2009). While this follows previous reports (Bak, 2003) and proves strategic given our focus on FLE, future studies should aim to disentangle the specific patterns characterizing ecological language processing in occipital, temporal, and parietal epilepsy cohorts separately. Indeed, the lack of DTI and rsFC alterations in PCE may partly reflect the conflation of heterogeneous patient profiles (Yu et al., 2009), as noted in a review (Leyden et al., 2015). Moreover, a recent study shown that white matter disruptions are more severe in FLE than in temporal lobe epilepsy (Lin Huan et al., 2020), suggesting that they may be better captured in the former.

## 5. Conclusion

Our study suggests that action discourse tasks, supported with structural and functional connectivity metrics, may reveal differential, ecological, and neurally grounded markers of FLE relative to PCE. These findings directly address strong calls to identify sensitive cognitive measures that discriminate among epilepsy subtypes. Further work along these lines can nurture a promising agenda at the crossing of neurology and cognitive neuroscience.

## Supplementary Material

1

## Figures and Tables

**Table 1:** Demographic and neuropsychological characteristics of the study samples.

	Group FLEpatients(*n* = 19)	Healthy controls(*n* = 19)	PCE patients(*n* = 20)	Statistics *p*-values	Effect size
Sex (F:M)	10:9	9:10	11:9	0.95^a^	0.01^d^
Handedness (R:L)	16:3	15:4	17:3	0.86^a^	0.01^d^
Age	27.47 (7.31)	31.15 (7.58)	28.65 (9.99)	0.39^b^	0.04^e^
Education	14.57 (1.67)	15.10 (1.72)	14.40 (1.66)	0.41 ^b^	0.03^e^
MoCA (overall)	25.94(1.96)	27.78(1.85)	26.35(2.23)	0.18^b^	0.07^e^
MoCA (attention subtest)	5.25 (0.45)	4.29 (0.78)	5.15 (0.57)	0.14^b^	0.08^e^
MoCA (language subtest)	4.36 (0.43)	4.93 (0.24)	3.98 (0.51)	0.21 ^b^	0.06^e^
IFS battery (overall)	23.36(1.78)	26.53(1.57)	25.04(1.89)	0.08^b^	0.10^e^
IFS (inhibition subtest)	3.34 (0.31)	3.75 (0.24)	2.93 (0.66)	0.25 ^b^	0.06^e^
IFS (memory subtest)	4.12 (0.52)	4.89 (0.81)	3.91 (0.55)	0.19 ^b^	0.05^e^
WASI	99.58(9.65)	108.68(10.25)	102.16(8.74)	0.29 ^b^	0.05^e^

Descriptive statistics are shown as mean (standard deviation);

a: *p*-value calculated via a chi-squared test.

b: *p*-value calculated via an independent measures ANOVA.

d: Cramer’s V.

e: Partial eta square. ANOVA; FLE: frontal lobe epilepsy; PCE: posterior cortex epilepsy; Handedness: Determined via the Edinburgh test.^32^ MoCA: Montreal Cognitive Assessment;^33^ IFS: INECO Frontal Screening;^34^ WASI: Weschler Abbreviated Scale of Intelligence. ^35^

ANOVA; FLE: frontal lobe epilepsy; PCE: posterior cortex epilepsy; Handedness: Determined via the Edinburgh test.^32^ MoCA: Montreal Cognitive Assessment;^33^ IFS: INECO Frontal Screening;^34^ WASI: Weschler Abbreviated Scale of Intelligence. ^35^

**Table 2:** Linguistic features of the texts.

	Action text 1	Action text 2	Neutral text 1	Neutraltext 2	*p*-values
Characters	941	908	976	934	0.47^#^
Words	207	203	204	199	0.98^#^
Nouns	48	48	44	43	0.93^#^
Adjectives	7	8	9	10	0.90^#^
Adverbs	6	8	8	8	0.94^#^
Verbs	32	32	32	32	1^#^
Action verbs	24	28	1	2	X (Braakman et al., 2013 Ma) = 9.94, *p* < 0.001. Tukey’s HSD tests showed that each NT differed from both ATs (*p* < 0.001), with no differences between NTs or ATs (all *p* > 0.58)
Non-action verbs	8	4	31	30	
Mean content word frequency	1.64 (0.08)	1.67 (0.08)	1.79 (0.08)	1.79 (0.08)	0.38*
Mean content word familiarity	6.17 (0.08)	6.00 (0.09)	6.28 (0.08)	6.23(0.09)	0.11*
Mean content word syllabic length	2.50 (0.08)	2.50 (0.09)	2.44 (0.09)	2.52 (0.09)	0.88*
Mean content word Orthographic length	5.95 (0.19)	5.70 (0.19)	6.03 (0.19)	5.93 (0.19)	0.63*
Mean content word imageability	5.17 (0.16)	5.27 (0.17)	4.96(0.16)	4.89 (0.17)	0.33*
Mean target verb frequency	1.08 (0.16)	1.48 (0.17)	1.10 (0.18)	1.43 (0.17)	0.22*
Mean target verb familiarity	5.61 (0.36)	6.20 (0.28)	6.23 (0.34)	6.09 (0.30)	0.58*
Mean target verb syllabic length	2.63 (0.19)	2.4 (0.18)	2.88 (0.19)	2.66 (0.17)	0.35*
Mean target verb orthographic length	6.00 (0.44)	6.50 (0.46)	7.44 (0.49)	6.55 (0.49)	0.19*
Mean target verb imageability	6.45 (0.42)	6.70 (0.44)	7.55 (0.46)	6.55 (0.46)	0.31*
Complex sentences (including subordinate clauses)	7	7	8	8	0.99^#^
Comprehensibility	4.5 (0.20)	4.10 (0.19)	4.38 (0.19)	4.18 (0.19)	0.44*
Coherence	4.0 (0.22)	3.52 (0.21)	4.00 (0.21)	3.73 (0.21)	0.32*
Grammatical Correctness	4.45 (0.18)	4.14 (0.17)	4.24 (0.17)	4.36 (0.17)	0.28*
Reading difficulty^a^	79.38	79.93	77.9	75.09	0.98
Readability^b^	Fairly Easy	Fairly easy	Fairly Easy	Fairly easy	-
Emotional valence (main effect of text)	33.38 (1.40)	33.54 (1.40)	33.33 (1.40)	33.23 (1.40)	0.99*
Arousal (main effect of text)	2.02 (0.12)	2.40 (0.12)	2.14 (0.12)	2.44 (0.12)	*F*(240,3) = 2.82, *p* = 0.04. Tukey’s HSD test (*DMS* = 0.43 *df* = 240) showed no differences among the for texts (all *p* > 0.05)
Number of voiced segments^c^	158	199	168	206	0.85^#^
Number of silence segments^c^	62	60	65	59	0.80^#^
Fundamental frequency (Hz)^c^	141.99 (25.82)	140.34 (26.62)	141.62 (23.96)	141.34 (26.88)	0.24*
Energy (dB)^c^	23.20 (3.99)	25.51 (3.64)	24.21 (4.07)	23.18 (3.84)	0.22*

a: Measured through the Szigriszt-Pazos Index.

b: measured through the Inflezs scale.

c: Measured by NeuroSpeech. (Orozco-Arroyave et al., 2018)

The hashtag (#) indicates *p*-values calculated with chi-squared test.

The asterisk (*) indicates *p*-values calculated with independent measures ANOVA, considering text as a factor.
