# Supplementary material for: Multimodal neurocognitive markers of frontal lobe epilepsy: Insights from ecological text processing
Source: Neuroimage. Author manuscript; Available in PMC 2021 Jul 15. (PMC8272524; doi:10.1016/j.neuroimage.2021.117998)
Supplement: 1 [file NIHMS1715917-supplement-1.docx]

**Supplementary material**

| 1. Supplementary data 1. Power estimation 2. Supplementary data 2. Full transcriptions and English translations 3. Supplementary data 3. DTI methods 4. Supplementary data 4. fMRI methods 5. Supplementary data 5. Machine learning analysis details 6. Supplementary data 6. Action task statistical analysis 7. Supplementary data 7. Neutral task statistical analysis 8. Supplementary data 8. DTI statistical analysis of local FA measures 9. Supplementary data 9. DTI statistical analysis of global FA measures 10. Supplementary data 10. Rs-fMRI statistical analysis 11. Supplementary data 11. Regression analysis details for DTI 12. Supplementary data 12. Regression analysis details for rs-fMRI 13. Supplementary data 13. Machine learning analysis without textual features 14. Supplementary data 14. Machine learning analysis with cognitive features |
| --- |

**1. Supplementary data 1. Power estimation**

Using G*Power 3.1,[^1^](#_ENREF_1) we performed a power estimation analysis for a 3x2 mixed-effects model, considering an alpha of .05, a large effect size of η^2^ = 0.25 (as previously reported for the same experimental task),[^2^](#_ENREF_2) and a power of 0.8. Results indicated that a total sample size of 42, considering the three groups, was enough to reach the estimated effects. Our actual sample size (*n* = 58) reaches a power of .92

**2. Supplementary data 2. Full transcriptions and English translations**

**2.1. Action text 1**

**Spanish original:** ¡La playa, el lugar preferido de Pedro en todo el universo! Pedro tomó a su hermano de la mano y corrieron hacia el mar. Ya en la orilla, se sacó la remera, los zapatos y los anteojos y se metió al agua. Sumergió la cabeza y buceó bajo las olas. Al llegar al fondo, Pedro agarró un caracol de abajo de un par de piedras. Un caracol exótico. Luego, su hermano nadó hasta él y lo retó a una carrera. ¡Era el momento de nadar hasta el muelle! Pedro nadaba adelante y... ¡ganó! Se sentía muy cansado. ¡Había nadado un montón! Luego, se acercó hasta el muelle y se agarró de una columna. Se subió de un salto. ¡Qué linda vista del horizonte! Era un hermoso atardecer. Mientras, su hermano seguía jugando en el mar. Pedro se levantó rápidamente y corrió hasta el otro lado del muelle. Luego, agarró una piedra y la arrojó hacia el agua con fuerza. Finalmente, su hermano salió del agua. Ambos se acercaron a un grupo de chicos que armaban castillos de arena y se agacharon para ayudarlos. Luego, todos caminaron hasta la casa de los hermanos, donde comieron una gran torta de chocolate. Fue una tarde muy divertida.

**English translation:** The beach, Pedro’s favorite place in the universe! Pedro took his brothers’ hand and ran towards the sea. In the rim, he took off his shirt, his shoes and glasses, and dove into the water. Submerged his head and swam under the waves. When reaching the bottom, Pedro took a snail from beneath some rocks. An exotic snail. Then, his brother swam towards him and challenged him for a race. It was time to swim until the dock! Pedro swam in front and won! He felt very tired by the long swim! Then, while walking towards the dock he hung from a column. He climbed jumping. What a pretty horizon view! It was a beautiful sunset. Meanwhile, his brothers continued to play in the sea. Pedro stood up quickly and ran to the other side of the docks. Then, he took a rock and threw it strongly to the water. Lastly, his brother went out of the water. Both of them joined a group of kids building sandcastles by helping them with its construction. Then, they walked together to the brother’s house, when they ate a big chocolate cake. It was a very funny evening.

**2.2. Action text 2**

**Spanish original:** Sábado por la tarde. ¡El momento favorito de Juancito en toda la semana! Tomó a sus padres de la mano y juntos corrieron hasta la plaza. Al lado de las hamacas, un grupo de niños aplaudía las piruetas de un colorido payaso. Juancito corrió velozmente hacia el lugar donde el payaso saltaba y bailaba sin cesar. Al terminar el espectáculo, el payaso escribió su nombre en el pavimento. ¡Qué sorpresa! ¡También se llamaba Juan! Luego, entre toda la muchedumbre, Juancito caminó hacia el lugar donde se sentaron sus padres. Abrazó a su padre con mucha fuerza y le jaló la camisa para que se levantara. ¡Era hora de jugar al fútbol! Juancito tomó la pelota y la puso en el pasto. Su padre se movía de izquierda a derecha, en posición de arquero. Juancito pateó y… ¡gol! Repentinamente salió el sol. Juancito se sacó el abrigo y lo apoyó en el asiento. Luego, su madre se acercó y le entregó un chocolate. Se lo comió de un bocado. Como siempre, al terminarlo, arrojó el paquete en el basurero. Había sido una intensa jornada. Se sentía muy cansado. Ya en la falda de su madre, mientras se limpiaba los restos de chocolate de la boca, se quedó dormido.

**English translation:** Saturday afternoon. Johnny’s favorite time of the week! He grabbed his parents by the hand and ran with them to the park. Next to the swings, a group of children applauded the antics of a colorful clown. Johnny ran quickly to the place where the clown jumped and danced non-stop. When the show was over, the clown wrote his name on the pavement. What surprise! He was also called Juan! Then, among the crowd, Johnny walked to the bench where his parents were sitting. He hugged his father with strength and pulled him by the shirt to have him stand up. It was time to play soccer! Johnny grabbed the ball and put it on the grass. His father moved from left to right, pretending to be a goalkeeper. Johnny kicked the ball and… goal! Suddenly, the sun appeared. Johnny removed his sweater and placed it on the bench. Then, his mother approached him and handed him a chocolate. He ate it in a single bite. As usual, upon finishing it, he threw the wrapping in the trash can. It had been a very intense day. He felt very tired. Once on his mother’s lap, while he wiped bits of chocolate off his mouth, he fell asleep.

**2.3. Neutral text 1**

**Spanish original:** ¡Qué felicidad, estaban de viaje! Clara estaba entusiasmada por haber invitado a su amiga con ella. Iban en el auto para el bosque y los paisajes eran hermosos. ¡Hora de disfrutar de las vacaciones! En un momento, Clara vio un animal en la ruta. No lo había visto nunca y tenía curiosidad. A su amiga, quien conducía, no le interesaban los animales, y menos uno raro en la ruta. Clara sabía que no era común y quería sacarle una foto. Al lado de la ruta, un grupo de autos detenidos fotografiaban y admiraban la belleza del animal. ¡Qué afortunados! Clara insistía, pero su amiga no se detenía por nada. Clara imaginaba historias de la criatura y se las contaba a su amiga, que se divertía. Luego de tanto insistir, Clara decidió disfrutar del paisaje a través de la ventanilla del auto. ¡Qué hermosa vista! Sacó un par de fotos a las montañas. Luego, impaciente, cerró los ojos y se quedó dormida. Se despertó un rato después. Sólo había dormido unos minutos. ¡Qué bien! En ese tiempo habían avanzado un montón. ¡Ya llegaban! Una hora más tarde, ya en el bosque, Clara disfrutaba del paisaje y del olor de las flores.

**English translation:** It was great! They were traveling! Clara was enthusiastic about inviting a friend to travel with her. They were driving a car into the forest and the views were beautiful. Time to enjoy vacations! In one moment, Clara saw an animal on the road. She had never seen it before and was curious about it. Her friend, who was driving, was not interested in animals and even less for an exotic one on the road. Clara knew that it was exotic and wanted to take a picture of it. Next to the road, a group of cars was stopped and his passengers were taking pictures and admiring the animal. How lucky! Clara insisted, but her friend would not stop. Clara imagined stories about the animal while she was telling them to her friend, who was having a good time. After insisting, Clara decided to enjoy the trip looking through the car window. What a beautiful sight! She took some pictures of the mountains. Then, impatiently, she closed her eyes and fall asleep. She woke up a little later. She just slept for some minutes. Great! During that time, they traveled a lot. They were almost there! One hour later, deep into the forest, Clara enjoyed the view and the smell of the flowers.

**2.4. Neutral text 2**

**Spanish original:** La noche recién comenzaba. Alberto estaba contento. ¡Gracias a Dios por los fines de semana! A unas pocas cuadras, la discoteca. Sus amigos lo esperaban allí y juntos compartirían un buen momento. Al cruzar la calle, Alberto leyó el nombre de la discoteca en un cartel: “Ni jefe ni reloj”. Siempre lo ponía de buen humor. Una vez adentro, lo enceguecieron las luces. Sintió calor y se encontró muy transpirado. Al lado del bar, un grupo de mujeres se entretenía con las historias de su amigo, Mario. Las muchachas se reían sin pausa junto a ese joven que bromeaba e inventaba personajes. Luego, entre toda la gente, Alberto reconoció a su novia, Elsa. Ella lo esperaba en una silla. Desde atrás, Alberto le preguntó si le gustaba la música. “¡Por supuesto!”, respondió Elsa. Aunque ella tenía sueño, Alberto le insistió para que lo acompañara a la pista. ¡Era hora de disfrutar la música! Como siempre, al decidirse, Elsa se olvidó la cartera en la silla. Alberto escuchó su canción favorita y se entusiasmó mucho. Elsa, fiel compañera, lo ayudó a recordar la letra. ¡Qué buen equipo! De regreso en su casa, mientras sentía el sudor y el cansancio en el cuerpo, se quedó dormido.

**English translation:** The night was just beginning. Albert was euphoric. Thank God for weekends! The disco was just a few blocks away. His friends were waiting for him there to have a good a time together. Upon crossing the street, Albert read the name of the disco on a sign: “No boss, no clock”. It always cheered him up. Once inside, he was blinded by lights. He felt the heat and found himself soaked in wet. Next to the bar, a group of women were entertained by the bantering of his friend, Mark. The girls were laughing non-stop with that young man who joked and invented characters. Then, among the crowd, Albert recognized his girlfriend, Elsa. She was waiting for him sitting on a chair. From behind, Albert asked her whether she liked the music. “Of course!,” replied Elsa. Though she was sleepy, Albert insisted that she joined him on the dancing floor. It was time to enjoy the music! As usual, upon deciding, Elsa forgot her purse on the chair. Albert heard his favorite song and was very excited. Elsa, ever the good sport, helped him remember the lyrics. What a good team! On the way back home, as he felt the sweat and tiredness all over his body, Albert fell asleep.

**3. Supplementary data 3. DTI methods details**

Diffusion-weighted images were obtained through a spin-echo echo-planar imaging sequence, with the following parameters: repetition time (TR) = 12000 ms, echo time (TE) = 68 ms, field of view = 224 mm, b-value = 0 or 1000 s/mm2, matrix 256 × 256, slice thickness = 3 mm, slice distance = 0 mm. A total of 50 slices were acquired with 64 diffusion directions, over a total scan time of 12 minutes. Images were first corrected for distortions caused by eddy currents,[^3^](#_ENREF_3) and the diffusion tensor was estimated through a linear regression.[^4^](#_ENREF_4) To obtain the final DTI images, the eigenvalues of the diffusion tensor were calculated by diagonalizing the tensor matrix. All post-processing steps were performed via FMRIB Software Library (FSL) tools, version 6.0.[^5^](#_ENREF_5)

As in previous works,[^6^](#_ENREF_6)^,^ [^7^](#_ENREF_7) WM tracts were characterized via the Tract-Based Spatial Statistics software toolbox.[^8^](#_ENREF_8) Local FA measures were used to pairwise compare WM integrity between the FLE patients, in which higher FA scores reflect tracts with higher white matter (WM) integrity.[^6^](#_ENREF_6) PCE patients, and controls via one-tailed two-sample t-tests using the FSL Randomise tool,[^5^](#_ENREF_5) performing 5000 permutations of Threshold-Free Cluster Enhancement. The analysis yielded significance maps corrected for multiple comparisons through the family-wise error (FWE) metric (p < .05). Global FA measures were also calculated for the correlation analyses (section 2.6) by averaging across the obtained skeletonized tracts. These maps were then parsed according to the John Hopkins ICBM DTI-based WM tract probability atlas,[^9^](#_ENREF_9) considering a total of 10 WM tracts,[^10^](#_ENREF_10) namely: forceps minor (Fmin), ATR, cingulate gyrus cingulum (CING), superior longitudinal fasciculus (SLF), inferior longitudinal fasciculus (ILF), corticospinal tract (CST), forceps major (Fmaj), uncinate fasciculus (UNC), hippocampal cingulum (CING-hipp), and inferior fronto-occipital fasciculus (IFOF).

**4. Supplementary data 4. fMRI methods details**

We used the following acquisition parameters: 50 slices acquired in ascending order, orientation parallel to the anterior and posterior commissures, voxel size = 3 x 3 x 3 mm^3^, repetition time (TR) = 2800 ms, echo time (TE) = 21 ms, flip angle = 90°, acquisition time = 15 minutes, number of volumes = 320.

Images were preprocessed on the Data Processing Assistant for Resting-State fMRI (DPARSF V.2.3)[^11^](#_ENREF_11) software, an open-access toolbox that generates automatic pipelines for analyzing resting-state fMRI data. For each preprocessing step, DPARSF called the Statistical Parametric Mapping (SPM 12**)**[^12^](#_ENREF_12) and the Resting-State fMRI Data Analysis Toolkit (REST V.1.7).[^13^](#_ENREF_13) Before preprocessing, the first five volumes of each subject’s resting-state session were discarded to ensure steady state magnetization. Images were then slice-time corrected (using as reference the middle slice of each volume) and aligned to the first scan of the session to correct for head movements. To reduce the effects of motion and physiological artifacts, six head-motion parameters, as well as WM and cerebrospinal fluid signals (CFS), were removed as nuisance variables. CFS and WM masks for this procedure were derived from the tissue segmentation of each subject’s T1 scan in native space. Next, functional images were normalized to the MNI space using the echo-planar imaging template from SPM [^14^](#_ENREF_14), and then they were smoothed with an 8-mm full-width half-maximum Gaussian kernel. Finally, data were band-pass filtered (0.01-0.08 Hz) given the relevance of slow frequencies in the analysis of resting-state networks .

RsFC for each network was calculated based on three seeds of 7x7x7 voxels, namely: a left, a right, and a bilateral seed, established via MNI coordinates. For the MN, seeds were placed in the M1 region (left: -32, -30, 68; right: 32, -30, 68), as reported in[^15^](#_ENREF_15). For the SemN, seeds were located in the ventral anterior temporal lobe (left: -51, 6, -39; right: 51, 6, -39).[^16^](#_ENREF_16) For the VN, seeds were placed in the primary visual area (V1) (left: -8, -92, 8; right: 8, -92, 8), as reported in[^17^](#_ENREF_17). Connectivity differences between group pairs (FLE patients vs. controls, PCE patients vs. controls, FLE vs. PCE patients) were calculated via one-tailed two-sample *t*-tests (*p* < .05, extent threshold = 50 voxels), corrected for multiple comparisons via the false discovery rate (FDR) metric [^18^](#_ENREF_18). For the correlation analyses, we averaged the obtained clusters from a two-tailed one-sample *t*-test in each group (*p* < .05, extent threshold = 50 voxels), FDR-corrected.[^19^](#_ENREF_19)

**5. Supplementary data 5. Machine learning analysis details**

We trained the model with all the set of normalized features (i.e., verb-related and circumstantial information outcomes in each text type, FA results for the 10 JHU atlas tracts, and results for each of the seeds in each of the rsFC networks). For the training phase in all our analyses, following best practices, we employed a *k*-fold cross-validation for hyper-parameter tuning.[^20^](#_ENREF_20) First, we ran a classifier to discriminate FLE patients from controls. Then, to test the specificity of predicted results, we examined the classification accuracy between PCE patients and controls, and then between FLE and PCE patients. To establish which features were most relevant in each classification scheme, we employed the feature importance analysis technique built in in our machine learning algorithm.[^21^](#_ENREF_21) We used a GBM classifier library called XGBoost [^21^](#_ENREF_21) (eXtreme Gradient Boosting) because of its high accuracy and robustness relative to other algorithms, tuning its hyper-parameters by Bayesian Optimization.[^22^](#_ENREF_22)^,^ [^23^](#_ENREF_23) GBMs are based on the gradient boosting technique, in which ensembles of decision trees iteratively attempt to correct the classification errors of their predecessors by minimizing a loss function (i.e., a function representing the difference between the estimated and true values) while pointing in the negative gradient direction [^24^](#_ENREF_24). The XGBoost classifier provides parallel computation tree boosting, enabling fast and accurate predictions which have proven successful in several fields;[^25-27^](#_ENREF_25) and also regularized boosting, helping to reduce overfitting and thus providing more generalizable results.[^27^](#_ENREF_27)^,^ [^28^](#_ENREF_28)

**6. Supplementary data 6. AT results**

**Table S1.** ANCOVA results for the ATs, controlling for MoCA and IFS.

|  | **Sum sq** | ***P*** |
| --- | --- | --- |
| MoCA | 22.1 | 0.54 |
| IFS | 8.6 | 0.70 |
| Group | 215.4 | 0.25 |
| Information type | 419.5 | 0.02 |
| Information type * MoCA | 438.7 | 0.01 |
| Information type * IFS | 10.7 | 0.70 |
| Information type * Group | 503.8 | 0.01 |

MoCA: Montreal Cognitive Assessment. IFS: INECO Frontal Screening. Group: Frontal lobe epilepsy, Posterior cortex epilepsy or Healthy control. Information type: verb-related information or circumstantial items.

**Table S2.** Post-hoc comparisons of the within-text analyses for the ATs, via Tukey’s HSD test.

| **Contrast** | **Cohen’s *d*** | ***T* ratio** | ***p*** |  |
| --- | --- | --- | --- | --- |
|  |  |  |  |  |
| Control,Circ – FLE,Circ | 0.57 | 1.95 | 0.12 |  |
| Control,Circ – PCE,Circ | 0.38 | 1.09 | 0.24 |  |
| Control,Circ – Control,Verb | 0.41 | -1.24 | 0.17 |  |
| Control,Circ – FLE,Verb | 0.38 | 0.98 | 0.25 |  |
| Control,Circ – PCE,Verb | 0.32 | 0.75 | 0.31 |  |
| FLE,Circ – PCE,Circ | 0.45 | 1.37 | 0.15 |  |
| FLE,Circ – Control,Verb | 0.39 | -1.19 | 0.23 |  |
| FLE,Circ – FLE,Verb | 0.95 | 4.21 | 0.01 |  |
| FLE,Circ – PCE,Verb | 0.31 | 0.74 | 0.32 |  |
| PCE,Circ – Control,Verb | 0.35 | 0.87 | 0.26 |  |
| PCE,Circ – FLE,Verb | 0.67 | 2.09 | 0.10 |  |
| PCE,Circ – PCE,Verb | 0.46 | 1.84 | 0.12 |  |
| Control,Verb – FLE,Verb | 0.85 | 4.05 | 0.03 |  |
| Control,Verb – PCE,Verb | 0.29 | 0.69 | 0.32 |  |
| FLE,Verb – PCE,Verb | 0.27 | -0.53 | 0.42 |  |
|  | | | | |
| Control: healthy controls; FLE: Frontal lobe epilepsy; PCE: Posterior cortex epilepsy; Verb: verb-related information; Circ: circumstantial information. | | | | |

**7. Supplementary data 7. NT results**

**Table S3.** ANCOVA rsulst for the NTs, controlling for MoCA and IFS.

|  | **Sum sq** | ***p*** |
| --- | --- | --- |
| MoCA | 0 | 0.98 |
| IFS | 23.2 | 0.58 |
| Group | 52.9 | 0.73 |
| Information type | 28.6 | 0.50 |
| Information type * MoCA | 6 | 0.92 |
| Information type * IFS | 133.9 | 0.15 |
| Information type * Group | 227.7 | 0.68 |

MoCA: Montreal Cognitive Assessment. IFS: INECO Frontal Screening. Group: Frontal lobe epilepsy, Posterior cortex epilepsy or Healthy control. Information type: verb-related information or circumstantial items.

**Table S4.** Post-hoc comparisons of the within-text analyses for the NTs via Tukey’s HSD test, controlling for MoCA and IFS.

| **Contrast** | **Cohen’s *d*** | ***T* ratio** | ***p*** |  |
| --- | --- | --- | --- | --- |
|  |  |  |  |  |
| Control,Circ – FLE,Circ | 0.05 | -0.12 | 0.65 |  |
| Control,Circ – PCE,Circ | 0.06 | 0.24 | 0.65 |  |
| Control,Circ – Control,Verb | 0.51 | 1.94 | 0.13 |  |
| Control,Circ – FLE,Verb | 0.38 | 0.96 | 0.26 |  |
| Control,Circ – PCE,Verb | 0.49 | 1.91 | 0.13 |  |
| FLE,Circ – PCE,Circ | 0.11 | 0.42 | 0.57 |  |
| FLE,Circ – Control,Verb | 0.08 | 0.29 | 0.62 |  |
| FLE,Circ – FLE,Verb | 0.14 | 0.43 | 0.57 |  |
| FLE,Circ – PCE,Verb | 0.19 | 0.75 | 0.38 |  |
| PCE,Circ – Control,Verb | 0.27 | 0.91 | 0.32 |  |
| PCE,Circ – FLE,Verb | 0.09 | 0.39 | 0.59 |  |
| PCE,Circ – PCE,Verb | 0.55 | 1.96 | 0.12 |  |
| Control,Verb – FLE,Verb | 0.21 | 0.89 | 0.36 |  |
| Control,Verb – PCE,Verb | 0.15 | 0.51 | 0.42 |  |
| FLE,Verb – PCE,Verb | 0.39 | 1.86 | 0.14 |  |
| MoCA: Montreal Cognitive Assessment. IFS: INECO Frontal Screening. Control: healthy controls; FLE: Frontal lobe epilepsy; PCE: Posterior cortex epilepsy; Verb: verb-related information; Circ: circumstantial information. | | | | |

**8. Supplementary data 8. DTI statistical analysis of local FA measures**

**Table S5.** Complete list of surviving voxels for the FLE patients < healthy controls contrast, in regions according to the John Hopkins white-matter tractography atlas.

| **JHU tract** | **Surviving voxels** | ***T score*** | ***p* (FWE-corrected)** |  |
| --- | --- | --- | --- | --- |
|  |  |  |  |  |
| Anterior thalamic radiations | 1264 | 5.27 | 0.03 |  |
| Uncinate fasciculus | 0 | 1.64 | 0.13 |  |
| Corticospinal tracts | 0 | 1.54 | 0.13 |  |
| Cingulum (hippocampus) | 0 | 1.21 | 0.26 |  |
| Cingulum (cingulate gyrus) | 0 | 0.85 | 0.32 |  |
| Superior longitudinal fasciculus | 0 | 0.84 | 0.38 |  |
| Inferior longitudinal fasciculus | 0 | 0.65 | 0.57 |  |
| Forceps minor | 0 | 0.36 | 0.57 |  |
| Inferior fronto-occipital fasciculus | 0 | 0.15 | 0.62 |  |
| Forceps major | 0 | 0.14 | 0.65 |  |
| JHU: John Hopkins University. | | | | |

**Table S6.** Complete list of surviving voxels for the healthy controls < FLE patients, in regions according to the John Hopkins white-matter tractography atlas.

| **JHU tract** | **Surviving voxels** | ***T score*** | ***p* (FWE-corrected)** |  |
| --- | --- | --- | --- | --- |
|  |  |  |  |  |
| Anterior thalamic radiations | 0 | 0.32 | 0.61 |  |
| Uncinate fasciculus | 0 | 0.25 | 0.57 |  |
| Corticospinal tracts | 0 | 0.24 | 0.59 |  |
| Cingulum (hippocampus) | 0 | 0.52 | 0.59 |  |
| Cingulum (cingulate gyrus) | 0 | 0.52 | 0.58 |  |
| Superior longitudinal fasciculus | 0 | 0.52 | 0.56 |  |
| Inferior longitudinal fasciculus | 0 | 0.19 | 0.52 |  |
| Forceps minor | 0 | 0.37 | 0.55 |  |
| Inferior fronto-occipital fasciculus | 0 | 0.48 | 0.53 |  |
| Forceps major | 0 | 0.45 | 0.53 |  |
| JHU: John Hopkins University. | | | | |

**Table S7.** Complete list of surviving voxels for the PCE patients < healthy controls contrast, in regions according to the John Hopkins white-matter tractography atlas.

| **JHU tract** | **Surviving voxels** | ***T score*** | ***p* (FWE-corrected)** |  |
| --- | --- | --- | --- | --- |
|  |  |  |  |  |
| Anterior thalamic radiations | 0 | 0.26 | 0.57 |  |
| Uncinate fasciculus | 0 | 0.17 | 0.56 |  |
| Corticospinal tracts | 0 | 0.19 | 0.51 |  |
| Cingulum (hippocampus) | 0 | 0.32 | 0.52 |  |
| Cingulum (cingulate gyrus) | 0 | 0.31 | 0.53 |  |
| Superior longitudinal fasciculus | 0 | 0.35 | 0.56 |  |
| Inferior longitudinal fasciculus | 0 | 0.11 | 0.55 |  |
| Forceps minor | 0 | 0.12 | 0.59 |  |
| Inferior fronto-occipital fasciculus | 0 | 0.42 | 0.62 |  |
| Forceps major | 0 | 0.17 | 0.53 |  |
| JHU: John Hopkins University. | | | | |

**Table S8.** Complete list of surviving voxels for the PCE patients > healthy controls contrast, in regions according to the John Hopkins white-matter tractography atlas.

| **JHU tract** | **Surviving voxels** | ***T score*** | ***p* (FWE-corrected)** |  |
| --- | --- | --- | --- | --- |
|  |  |  |  |  |
| Anterior thalamic radiations | 0 | 0.32 | 0.58 |  |
| Uncinate fasciculus | 0 | 0.52 | 0.56 |  |
| Corticospinal tracts | 0 | 0.14 | 0.57 |  |
| Cingulum (hippocampus) | 0 | 0.12 | 0.59 |  |
| Cingulum (cingulate gyrus) | 0 | 0.22 | 0.57 |  |
| Superior longitudinal fasciculus | 0 | 0.18 | 0.61 |  |
| Inferior longitudinal fasciculus | 0 | 0.45 | 0.62 |  |
| Forceps minor | 0 | 0.14 | 0.51 |  |
| Inferior fronto-occipital fasciculus | 0 | 0.18 | 0.55 |  |
| Forceps major | 0 | 0.19 | 0.61 |  |
| JHU: John Hopkins University. | | | | |

**Table S9.** Complete list of surviving voxels for the FLE patients < PCE patients, in regions according to the John Hopkins white-matter tractography atlas.

| **JHU tract** | **Surviving voxels** | ***T score*** | ***p* (FWE-corrected)** |  |
| --- | --- | --- | --- | --- |
|  |  |  |  |  |
| Anterior thalamic radiations | 0 | 0.21 | 0.51 |  |
| Uncinate fasciculus | 0 | 0.34 | 0.57 |  |
| Corticospinal tracts | 0 | 0.47 | 0.56 |  |
| Cingulum (hippocampus) | 0 | 0.37 | 0.61 |  |
| Cingulum (cingulate gyrus) | 0 | 0.28 | 0.54 |  |
| Superior longitudinal fasciculus | 0 | 0.19 | 0.52 |  |
| Inferior longitudinal fasciculus | 0 | 0.11 | 0.58 |  |
| Forceps minor | 0 | 0.22 | 0.56 |  |
| Inferior fronto-occipital fasciculus | 0 | 0.21 | 0.58 |  |
| Forceps major | 0 | 0.19 | 0.61 |  |
| JHU: John Hopkins University. | | | | |

**Table S10.** Complete list of surviving voxels for the FLE patients > PCE patients contrast, in regions according to the John Hopkins white-matter tractography atlas.

| **JHU tract** | **Surviving voxels** | ***T score*** | ***p* (FWE-corrected)** |  |
| --- | --- | --- | --- | --- |
|  |  |  |  |  |
| Anterior thalamic radiations | 0 | 0.34 | 0.57 |  |
| Uncinate fasciculus | 0 | 0.12 | 0.51 |  |
| Corticospinal tracts | 0 | 0.28 | 0.56 |  |
| Cingulum (hippocampus) | 0 | 0.13 | 0.54 |  |
| Cingulum (cingulate gyrus) | 0 | 0.18 | 0.59 |  |
| Superior longitudinal fasciculus | 0 | 0.15 | 0.61 |  |
| Inferior longitudinal fasciculus | 0 | 0.26 | 0.59 |  |
| Forceps minor | 0 | 0.13 | 0.54 |  |
| Inferior fronto-occipital fasciculus | 0 | 0.39 | 0.61 |  |
| Forceps major | 0 | 0.34 | 0.53 |  |
| JHU: John Hopkins University. | | | | |

**9. Supplementary data 9. DTI statistical analysis of global FA measures**

**Table S11.** Global FA measures (averaged in JHU atlas tracts) for the FLE patients < healthy controls *t*-test.

| **JHU tract** | **Cohen’s *d*** | ***T score*** | ***p* (FDR-corrected)** |  |
| --- | --- | --- | --- | --- |
|  |  |  |  |  |
| Anterior thalamic radiations | 0.83 | 2.45 | 0.03 |  |
| Uncinate fasciculus | 0.71 | 1.65 | 0.14 |  |
| Corticospinal tracts | 0.65 | 1.32 | 0.15 |  |
| Cingulum (hippocampus) | 0.56 | 1.12 | 0.18 |  |
| Cingulum (cingulate gyrus) | 0.24 | 0.82 | 0.23 |  |
| Superior longitudinal fasciculus | 0.23 | 0.79 | 0.26 |  |
| Inferior longitudinal fasciculus | 0.12 | 0.62 | 0.32 |  |
| Forceps minor | 0.11 | 0.41 | 0.34 |  |
| Inferior fronto-occipital fasciculus | 0.09 | 0.32 | 0.35 |  |
| Forceps major | 0.07 | 0.15 | 0.37 |  |
| JHU: John Hopkins University. | | | | |

**Table S12.** Global FA measures (averaged in JHU atlas tracts) for the PCE patients < healthy controls *t*-test.

| **JHU tract** | **Cohen’s *d*** | ***T score*** | ***p* (FDR-corrected)** |  |
| --- | --- | --- | --- | --- |
|  |  |  |  |  |
| Anterior thalamic radiations | 0.12 | 1.56 | 0.32 |  |
| Uncinate fasciculus | 0.32 | 0.91 | 0.49 |  |
| Corticospinal tracts | 0.15 | 1.32 | 0.35 |  |
| Cingulum (hippocampus) | 0.13 | 1.42 | 0.35 |  |
| Cingulum (cingulate gyrus) | 0.24 | 1.09 | 0.41 |  |
| Superior longitudinal fasciculus | 0.13 | 1.41 | 0.34 |  |
| Inferior longitudinal fasciculus | 0.27 | 0.92 | 0.45 |  |
| Forceps minor | 0.31 | 0.91 | 0.49 |  |
| Inferior fronto-occipital fasciculus | 0.24 | 0.96 | 0.42 |  |
| Forceps major | 0.16 | 1.15 | 0.36 |  |
| JHU: John Hopkins University. | | | | |

**Table S13.** Global FA measures (averaged in JHU atlas tracts) for the FLE patients < PCE patients *t*-test.

| **JHU tract** | **Cohen’s *d*** | ***T score*** | ***p* (FDR-corrected)** |  |
| --- | --- | --- | --- | --- |
|  |  |  |  |  |
| Anterior thalamic radiations | 0.65 | 2.01 | 0.09 |  |
| Uncinate fasciculus | 0.56 | 1.91 | 0.15 |  |
| Corticospinal tracts | 0.49 | 1.85 | 0.19 |  |
| Cingulum (hippocampus) | 0.39 | 1.26 | 0.56 |  |
| Cingulum (cingulate gyrus) | 0.35 | 1.19 | 0.68 |  |
| Superior longitudinal fasciculus | 0.29 | 1.09 | 0.65 |  |
| Inferior longitudinal fasciculus | 0.31 | 1.11 | 0.64 |  |
| Forceps minor | 0.21 | 0.93 | 0.78 |  |
| Inferior fronto-occipital fasciculus | 0.25 | 1.05 | 0.63 |  |
| Forceps major | 0.29 | 1.09 | 0.65 |  |
| JHU: John Hopkins University. | | | | |

**Table S14.** Global FA measures (averaged in JHU atlas tracts) for the FLE patients > healthy controls *t*-test.

| **JHU tract** | **Cohen’s *d*** | ***T score*** | ***p* (FDR-corrected)** |  |
| --- | --- | --- | --- | --- |
|  |  |  |  |  |
| Anterior thalamic radiations | 0.24 | 1.29 | 0.56 |  |
| Uncinate fasciculus | 0.27 | 1.24 | 0.58 |  |
| Corticospinal tracts | 0.32 | 1.42 | 0.60 |  |
| Cingulum (hippocampus) | 0.31 | 1.39 | 0.53 |  |
| Cingulum (cingulate gyrus) | 0.35 | 1.47 | 0.63 |  |
| Superior longitudinal fasciculus | 0.32 | 1.42 | 0.55 |  |
| Inferior longitudinal fasciculus | 0.14 | 1.15 | 0.53 |  |
| Forceps minor | 0.26 | 1.32 | 0.53 |  |
| Inferior fronto-occipital fasciculus | 0.13 | 1.14 | 0.54 |  |
| Forceps major | 0.21 | 1.25 | 0.59 |  |
| JHU: John Hopkins University. | | | | |

**Table S15.** Global FA measures (averaged in JHU atlas tracts) for the PCE patients > healthy controls *t*-test.

| **JHU tract** | **Cohen’s *d*** | ***T score*** | ***p* (FDR-corrected)** |  |
| --- | --- | --- | --- | --- |
|  |  |  |  |  |
| Anterior thalamic radiations | 0.16 | 1.18 | 0.56 |  |
| Uncinate fasciculus | 0.34 | 1.45 | 0.62 |  |
| Corticospinal tracts | 0.24 | 1.29 | 0.58 |  |
| Cingulum (hippocampus) | 0.28 | 1.35 | 0.61 |  |
| Cingulum (cingulate gyrus) | 0.33 | 1.43 | 0.55 |  |
| Superior longitudinal fasciculus | 0.25 | 1.31 | 0.57 |  |
| Inferior longitudinal fasciculus | 0.13 | 1.14 | 0.58 |  |
| Forceps minor | 0.32 | 1.42 | 0.55 |  |
| Inferior fronto-occipital fasciculus | 0.37 | 1.57 | 0.55 |  |
| Forceps major | 0.14 | 1.15 | 0.59 |  |
| JHU: John Hopkins University. | | | | |

**Table S16.** Global FA measures (averaged in JHU atlas tracts) for the FLE patients > PCE patients *t*-test.

| **JHU tract** | **Cohen’s *d*** | ***T score*** | ***p* (FDR-corrected)** |  |
| --- | --- | --- | --- | --- |
|  |  |  |  |  |
| Anterior thalamic radiations | 0.18 | 1.21 | 0.55 |  |
| Uncinate fasciculus | 0.14 | 1.11 | 0.55 |  |
| Corticospinal tracts | 0.32 | 1.42 | 0.57 |  |
| Cingulum (hippocampus) | 0.11 | 1.12 | 0.55 |  |
| Cingulum (cingulate gyrus) | 0.36 | 1.48 | 0.60 |  |
| Superior longitudinal fasciculus | 0.25 | 1.31 | 0.58 |  |
| Inferior longitudinal fasciculus | 0.28 | 1.35 | 0.53 |  |
| Forceps minor | 0.13 | 1.14 | 0.57 |  |
| Inferior fronto-occipital fasciculus | 0.19 | 1.22 | 0.56 |  |
| Forceps major | 0.28 | 1.35 | 0.60 |  |
| JHU: John Hopkins University. | | | | |
|  | | | | |

**10. Supplementary data 10. Rs-fMRI statistical analysis**

**Table S17.** Rs-fMRI results for the FLE patients < healthy controls contrast using the wSDM measure (FDR-corrected *p* < .05).

| **Seed** | **Tresholded**  **cluster size** | | **Brain regions** | | **Cluster peak** | | | | | **Coordinates** | | | |
| --- | --- | --- | --- | --- | --- | --- | --- | --- | --- | --- | --- | --- | --- |
|  |  |  |  |  | ***T*** | | ***p*** | | | ***x*** | ***y*** | | ***z*** |
| Bilateral M1 | 816 | | Left parietal operculum (427 voxels)  Left supramarginal gyrus (389 voxels) | | 3.58 | | < 0.001 | | | -50 | -42 | | 24 |
|  |  |  |  |  |  |  |  |  |  |  |  |  |  |
| Left M1 | 0 |  | |  | |  | |  |  | | |  |  |
| Right M1 | 0 |  | |  | |  | |  |  | | |  |  |
| Bilateral vATL | 0 |  | |  | |  | |  |  | | |  |  |
| Left vATL | 0 |  | |  | |  | |  |  | | |  |  |
| Right vATL | 0 |  | |  | |  | |  |  | | |  |  |
| Bilateral V1 | 0 |  | |  | |  | |  |  | | |  |  |
| Left V1 | 0 |  | |  | |  | |  |  | | |  |  |
| Right V1 | 0 |  | |  | |  | |  |  | | |  |  |

M1: primary motor cortex, vATL: ventral anterior temporal lobe, V1: primary visual cortex.

No other rsFC comparison (i.e., FLE patients > healthy controls, PCE patients < healthy controls, PCE patients > healthy controls, FLE patients < PCE patients, FLE patients > PCE patients) revealed surviving clusters for the MN, the SemN, or the VN in any seed.

**11. Supplementary data 11. Regression analysis details for DTI**

**Table S18.** Correlations between white matter tracts’ FA and verb-related information in the ATs, for the FLE group.

| **JHU tract** | **Pearson’s *r*** | ***p* (FDR-corrected)** | |  |  |
| --- | --- | --- | --- | --- | --- |
|  |  |  | |  |  |
| Anterior thalamic radiations | 0.869 | 0.03 | |  |  |
| Uncinate fasciculus | -0.052 | 0.38 | |  |  |
| Corticospinal tracts | -0.061 | 0.32 | |  |  |
| Cingulum (hippocampus) | 0.078 | 0.25 | |  |  |
| Cingulum (cingulate gyrus) | 0.096 | 0.20 | |  |  |
| Superior longitudinal fasciculus | 0.116 | 0.17 | |  |  |
| Inferior longitudinal fasciculus | 0.116 | 0.17 | |  |  |
| Forceps minor | 0.027 | 0.74 | |  |  |
| Inferior fronto-occipital fasciculus | -0.045 | 0.44 | |  |  |
| Forceps major | -0.043 | 0.46 | |  |  |
|  | | |  | | |
| JHU: John Hopkins University. | | | | |  |

**Table S19.** Correlations between white matter tracts’ FA and circumstantial information in the ATs, for the FLE group.

| **JHU tract** | **Pearson’s *r*** | ***p* (FDR-corrected)** |  |
| --- | --- | --- | --- |
|  |  |  |  |
| Anterior thalamic radiations | -0.105 | 0.55 |  |
| Uncinate fasciculus | -0.103 | 0.10 |  |
| Corticospinal tracts | -0.082 | 0.19 |  |
| Cingulum (hippocampus) | -0.107 | 0.18 |  |
| Cingulum (cingulate gyrus) | 0.034 | 0.58 |  |
| Superior longitudinal fasciculus | -0.109 | 0.18 |  |
| Inferior longitudinal fasciculus | -0.077 | 0.25 |  |
| Forceps minor | -0.118 | 0.16 |  |
| Inferior fronto-occipital fasciculus | -0.109 | 0.18 |  |
| Forceps major | 0.085 | 0.23 |  |
| JHU: John Hopkins University. | | | |

**Table S20.** Correlations between white matter tracts’ FA and verb-related information in the NTs, for the FLE group.

| **JHU tract** | **Pearson’s *r*** | ***p* (FDR-corrected)** |  |
| --- | --- | --- | --- |
|  |  |  |  |
| Anterior thalamic radiations | 0.004 | 0.37 |  |
| Uncinate fasciculus | 0.078 | 0.25 |  |
| Corticospinal tracts | 0.084 | 0.23 |  |
| Cingulum (hippocampus) | -0.086 | 0.23 |  |
| Cingulum (cingulate gyrus) | -0.038 | 0.52 |  |
| Superior longitudinal fasciculus | -0.009 | 0.22 |  |
| Inferior longitudinal fasciculus | 0.086 | 0.23 |  |
| Forceps minor | 0.093 | 0.21 |  |
| Inferior fronto-occipital fasciculus | 0.047 | 0.42 |  |
| Forceps major | 0.068 | 0.29 |  |
| JHU: John Hopkins University. | | | |

**Table S21.** Correlations between white matter tracts’ FA and circumstantial information in the NTs, for the FLE group.

| **JHU tract** | **Pearson’s *r*** | ***p* (FDR-corrected)** |  |
| --- | --- | --- | --- |
|  |  |  |  |
| Anterior thalamic radiations | -0.031 | 0.34 |  |
| Uncinate fasciculus | 0.062 | 0.32 |  |
| Corticospinal tracts | 0.056 | 0.35 |  |
| Cingulum (hippocampus) | -0.108 | 0.18 |  |
| Cingulum (cingulate gyrus) | -0.045 | 0.44 |  |
| Superior longitudinal fasciculus | 0.122 | 0.16 |  |
| Inferior longitudinal fasciculus | 0.111 | 0.18 |  |
| Forceps minor | -0.123 | 0.16 |  |
| Inferior fronto-occipital fasciculus | 0.055 | 0.36 |  |
| Forceps major | -0.117 | 0.17 |  |
| JHU: John Hopkins University. | | | |

**Table S22.** Correlations between white matter tracts’ FA and verb-related information in the ATs, for the healthy control group.

| **JHU tract** | **Pearson’s *r*** | ***p* (FDR-corrected)** |  |
| --- | --- | --- | --- |
|  |  |  |  |
| Anterior thalamic radiations | -0.036 | 0.35 |  |
| Uncinate fasciculus | 0.012 | 0.66 |  |
| Corticospinal tracts | 0.107 | 0.18 |  |
| Cingulum (hippocampus) | -0.041 | 0.48 |  |
| Cingulum (cingulate gyrus) | -0.097 | 0.20 |  |
| Superior longitudinal fasciculus | 0.041 | 0.48 |  |
| Inferior longitudinal fasciculus | 0.054 | 0.37 |  |
| Forceps minor | 0.047 | 0.13 |  |
| Inferior fronto-occipital fasciculus | -0.059 | 0.15 |  |
| Forceps major | -0.113 | 0.06 |  |
| JHU: John Hopkins University. | | | |

**Table S23.** Correlations between white matter tracts’ FA and circumstantial information in the ATs, for the healthy control group.

| **JHU tract** | **Pearson’s *r*** | ***p* (FDR-corrected)** |  |
| --- | --- | --- | --- |
|  |  |  |  |
| Anterior thalamic radiations | -0.067 | 0.11 |  |
| Uncinate fasciculus | -0.092 | 0.16 |  |
| Corticospinal tracts | 0.114 | 0.23 |  |
| Cingulum (hippocampus) | 0.08 | 0.42 |  |
| Cingulum (cingulate gyrus) | 0.078 | 0.27 |  |
| Superior longitudinal fasciculus | -0.088 | 0.11 |  |
| Inferior longitudinal fasciculus | -0.105 | 0.19 |  |
| Forceps minor | 0.031 | 0.66 |  |
| Inferior fronto-occipital fasciculus | -0.038 | 0.52 |  |
| Forceps major | -0.098 | 0.13 |  |
| JHU: John Hopkins University. | | | |

**Table S24.** Correlations between white matter tracts’ FA and verb-related information in the NTs, for the healthy control group.

| **JHU tract** | **Pearson’s *r*** | ***p* (FDR-corrected)** |  |
| --- | --- | --- | --- |
|  |  |  |  |
| Anterior thalamic radiations | 0.068 | 0.06 |  |
| Uncinate fasciculus | -0.049 | 0.40 |  |
| Corticospinal tracts | -0.097 | 0.20 |  |
| Cingulum (hippocampus) | 0.082 | 0.24 |  |
| Cingulum (cingulate gyrus) | -0.035 | 0.57 |  |
| Superior longitudinal fasciculus | -0.019 | 0.15 |  |
| Inferior longitudinal fasciculus | 0.063 | 0.31 |  |
| Forceps minor | 0.037 | 0.54 |  |
| Inferior fronto-occipital fasciculus | 0.051 | 0.39 |  |
| Forceps major | 0.019 | 0.15 |  |
| JHU: John Hopkins University. | | | |

**Table S25.** Correlations between white matter tracts’ FA and circumstantial information in the NTs, for the healthy control group.

| **JHU tract** | **Pearson’s *r*** | ***p* (FDR-corrected)** |  |
| --- | --- | --- | --- |
|  |  |  |  |
| Anterior thalamic radiations | -0.041 | 0.48 |  |
| Uncinate fasciculus | -0.092 | 0.21 |  |
| Corticospinal tracts | -0.122 | 0.16 |  |
| Cingulum (hippocampus) | 0.076 | 0.26 |  |
| Cingulum (cingulate gyrus) | -0.063 | 0.31 |  |
| Superior longitudinal fasciculus | 0.096 | 0.20 |  |
| Inferior longitudinal fasciculus | -0.095 | 0.21 |  |
| Forceps minor | -0.061 | 0.32 |  |
| Inferior fronto-occipital fasciculus | 0.092 | 0.21 |  |
| Forceps major | -0.113 | 0.17 |  |
| JHU: John Hopkins University. | | | |

**Table S26.** Correlations between white matter tracts’ FA and verb-related information in the ATs, for the PCE group.

| **JHU tract** | **Pearson’s *r*** | ***p* (FDR-corrected)** |  |
| --- | --- | --- | --- |
|  |  |  |  |
| Anterior thalamic radiations | 0.026 | 0.36 |  |
| Uncinate fasciculus | 0.094 | 0.21 |  |
| Corticospinal tracts | -0.058 | 0.34 |  |
| Cingulum (hippocampus) | 0.013 | 0.53 |  |
| Cingulum (cingulate gyrus) | 0.122 | 0.16 |  |
| Superior longitudinal fasciculus | -0.052 | 0.38 |  |
| Inferior longitudinal fasciculus | -0.05 | 0.43 |  |
| Forceps minor | -0.034 | 0.58 |  |
| Inferior fronto-occipital fasciculus | -0.064 | 0.31 |  |
| Forceps major | -0.044 | 0.45 |  |
| JHU: John Hopkins University. | | | |

**Table S27.** Correlations between white matter tracts’ FA and circumstantial information in the ATs, for the PCE group.

| **JHU tract** | **Pearson’s *r*** | ***p* (FDR-corrected)** |  |
| --- | --- | --- | --- |
|  |  |  |  |
| Anterior thalamic radiations | 0.033 | 0.60 |  |
| Uncinate fasciculus | 0.106 | 0.18 |  |
| Corticospinal tracts | 0.104 | 0.19 |  |
| Cingulum (hippocampus) | -0.007 | 0.85 |  |
| Cingulum (cingulate gyrus) | -0.022 | 0.90 |  |
| Superior longitudinal fasciculus | -0.07 | 0.28 |  |
| Inferior longitudinal fasciculus | -0.029 | 0.68 |  |
| Forceps minor | -0.105 | 0.19 |  |
| Inferior fronto-occipital fasciculus | 0.098 | 0.20 |  |
| Forceps major | 0.038 | 0.52 |  |
| JHU: John Hopkins University. | | | |

**Table S28.** Correlations between white matter tracts’ FA and verb-related information in the NTs, for the PCE group.

| **JHU tract** | **Pearson’s *r*** | ***p* (FDR-corrected)** |  |
| --- | --- | --- | --- |
|  |  |  |  |
| Anterior thalamic radiations | -0.074 | 0.27 |  |
| Uncinate fasciculus | 0.088 | 0.22 |  |
| Corticospinal tracts | 0.035 | 0.57 |  |
| Cingulum (hippocampus) | 0.104 | 0.19 |  |
| Cingulum (cingulate gyrus) | 0.118 | 0.16 |  |
| Superior longitudinal fasciculus | 0.066 | 0.30 |  |
| Inferior longitudinal fasciculus | 0.057 | 0.35 |  |
| Forceps minor | -0.051 | 0.39 |  |
| Inferior fronto-occipital fasciculus | -0.041 | 0.52 |  |
| Forceps major | -0.018 | 0.81 |  |
| JHU: John Hopkins University. | | | |

**Table S29.** Correlations between white matter tracts’ FA and circumstantial information in the NTs, for the PCE group.

| **JHU tract** | **Pearson’s *r*** | ***p* (FDR-corrected)** |  |
| --- | --- | --- | --- |
|  |  |  |  |
| Anterior thalamic radiations | 0.056 | 0.35 |  |
| Uncinate fasciculus | -0.033 | 0.64 |  |
| Corticospinal tracts | -0.117 | 0.17 |  |
| Cingulum (hippocampus) | 0.119 | 0.16 |  |
| Cingulum (cingulate gyrus) | 0.079 | 0.25 |  |
| Superior longitudinal fasciculus | -0.063 | 0.31 |  |
| Inferior longitudinal fasciculus | -0.056 | 0.35 |  |
| Forceps minor | -0.081 | 0.24 |  |
| Inferior fronto-occipital fasciculus | -0.096 | 0.20 |  |
| Forceps major | 0.014 | 0.42 |  |
| JHU: John Hopkins University. | | | |

**12. Supplementary data 12. Regression analysis details for rs-fMRI**

**Table S30.** Correlations between the wSDM measure and the verb-related information in the ATs, for the FLE group.

| **Seed** | **Pearson’s *r*** | ***p* (FDR-corrected)** |
| --- | --- | --- |
|  |  |  |
| Bilateral M1 | 0.707 | 0.04 |
| Left M1 | -0.065 | 0.30 |
| Right M1 | -0.093 | 0.21 |
| Bilateral vATL | 0.056 | 0.35 |
| Left vATL | 0.101 | 0.19 |
| Right vATL | -0.121 | 0.16 |
| Bilateral V1 | -0.109 | 0.18 |
| Left V1 | 0.031 | 0.64 |
| Right V1 | 0.093 | 0.22 |

M1: primary motor cortex, vATL: ventral anterior temporal lobe, V1: primary visual cortex.

**Table S31.** Correlations between the wSDM measure and the circumstantial information in the ATs, for the FLE group.

| **Seed** | **Pearson’s *r*** | ***p* (FDR-corrected)** |
| --- | --- | --- |
|  |  |  |
| Bilateral M1 | 0.041 | 0.39 |
| Left M1 | -0.091 | 0.21 |
| Right M1 | 0.036 | 0.55 |
| Bilateral vATL | -0.011 | 0.81 |
| Left vATL | 0.087 | 0.22 |
| Right vATL | -0.068 | 0.29 |
| Bilateral V1 | -0.121 | 0.16 |
| Left V1 | 0.013 | 0.53 |
| Right V1 | -0.124 | 0.16 |

M1: primary motor cortex, vATL: ventral anterior temporal lobe, V1: primary visual cortex.

**Table S32.** Correlations between the wSDM measure and the verb-related information in the ATs, for the FLE group.

| **Seed** | **Pearson’s *r*** | ***p* (FDR-corrected)** |
| --- | --- | --- |
|  |  |  |
| Bilateral M1 | -0.019 | 0.44 |
| Left M1 | -0.072 | 0.27 |
| Right M1 | 0.056 | 0.35 |
| Bilateral vATL | 0.027 | 0.74 |
| Left vATL | -0.109 | 0.18 |
| Right vATL | 0.058 | 0.34 |
| Bilateral V1 | 0.038 | 0.52 |
| Left V1 | -0.091 | 0.21 |
| Right V1 | 0.117 | 0.17 |

M1: primary motor cortex, vATL: ventral anterior temporal lobe, V1: primary visual cortex.

**Table S33.** Correlations between the wSDM measure and the circumstantial information in the NTs, for the FLE group.

| **Seed** | **Pearson’s *r*** | ***p* (FDR-corrected)** |
| --- | --- | --- |
|  |  |  |
| Bilateral M1 | -0.017 | 0.45 |
| Left M1 | -0.036 | 0.55 |
| Right M1 | -0.034 | 0.58 |
| Bilateral vATL | 0.101 | 0.19 |
| Left vATL | 0.122 | 0.16 |
| Right vATL | 0.092 | 0.21 |
| Bilateral V1 | 0.031 | 0.64 |
| Left V1 | 0.089 | 0.22 |
| Right V1 | -0.066 | 0.30 |

M1: primary motor cortex, vATL: ventral anterior temporal lobe, V1: primary visual cortex.

**Table S34.** Correlations between the wSDM measure and the verb-related information for in the ATs, for the healthy control group.

| **Seed** | **Pearson’s *r*** | ***p* (FDR-corrected)** |
| --- | --- | --- |
|  |  |  |
| Bilateral M1 | 0.022 | 0.90 |
| Left M1 | 0.036 | 0.55 |
| Right M1 | -0.106 | 0.18 |
| Bilateral vATL | 0.035 | 0.57 |
| Left vATL | -0.123 | 0.16 |
| Right vATL | -0.089 | 0.22 |
| Bilateral V1 | 0.094 | 0.21 |
| Left V1 | 0.017 | 0.97 |
| Right V1 | 0.079 | 0.25 |

M1: primary motor cortex, vATL: ventral anterior temporal lobe, V1: primary visual cortex.

**Table S35.** Correlations between the wSDM measure and the circumstantial information in the ATs, for the healthy control group.

| **Seed** | **Pearson’s *r*** | ***p* (FDR-corrected)** |
| --- | --- | --- |
|  |  |  |
| Bilateral M1 | 0.027 | 0.17 |
| Left M1 | 0.095 | 0.12 |
| Right M1 | 0.082 | 0.24 |
| Bilateral vATL | -0.018 | 0.81 |
| Left vATL | -0.049 | 0.40 |
| Right vATL | -0.025 | 0.84 |
| Bilateral V1 | -0.069 | 0.28 |
| Left V1 | 0.048 | 0.41 |
| Right V1 | -0.062 | 0.32 |

M1: primary motor cortex, vATL: ventral anterior temporal lobe, V1: primary visual cortex.

**Table S36.** Correlations between the wSDM measure and the verb-related information in the NTs, for the healthy control group.

| **Seed** | **Pearson’s *r*** | ***p* (FDR-corrected)** |
| --- | --- | --- |
|  |  |  |
| Bilateral M1 | 0.116 | 0.17 |
| Left M1 | -0.09 | 0.22 |
| Right M1 | -0.017 | 0.97 |
| Bilateral vATL | -0.101 | 0.19 |
| Left vATL | 0.11 | 0.18 |
| Right vATL | 0.121 | 0.16 |
| Bilateral V1 | -0.029 | 0.68 |
| Left V1 | -0.002 | 0.98 |
| Right V1 | 0.035 | 0.57 |

M1: primary motor cortex, vATL: ventral anterior temporal lobe, V1: primary visual cortex.

**Table S37.** Correlations between the wSDM measure and the circumstantial information in the NTs, for the healthy control group.

| **Seed** | **Pearson’s *r*** | ***p* (FDR-corrected)** |
| --- | --- | --- |
|  |  |  |
| Bilateral M1 | 0.021 | 0.95 |
| Left M1 | 0.044 | 0.45 |
| Right M1 | 0.076 | 0.26 |
| Bilateral vATL | 0.047 | 0.42 |
| Left vATL | -0.026 | 0.76 |
| Right vATL | 0.029 | 0.68 |
| Bilateral V1 | -0.103 | 0.19 |
| Left V1 | 0.09 | 0.22 |
| Right V1 | 0.116 | 0.17 |

M1: primary motor cortex, vATL: ventral anterior temporal lobe, V1: primary visual cortex.

**Table S38.** Correlations between the wSDM measure and the verb-related information in the ATs, for the PCE group.

| **Seed** | **Pearson’s *r*** | ***p* (FDR-corrected)** |
| --- | --- | --- |
|  |  |  |
| Bilateral M1 | -0.024 | 0.83 |
| Left M1 | -0.079 | 0.25 |
| Right M1 | -0.006 | 0.99 |
| Bilateral vATL | 0.074 | 0.27 |
| Left vATL | 0.102 | 0.19 |
| Right vATL | -0.066 | 0.30 |
| Bilateral V1 | -0.069 | 0.28 |
| Left V1 | -0.046 | 0.43 |
| Right V1 | -0.004 | 0.99 |

M1: primary motor cortex, vATL: ventral anterior temporal lobe, V1: primary visual cortex.

**Table S39.** Correlations between the wSDM measure and the circumstantial information in the ATs, for the PCE group.

| **Seed** | **Pearson’s *r*** | ***p* (FDR-corrected)** |
| --- | --- | --- |
|  |  |  |
| Bilateral M1 | 0.106 | 0.18 |
| Left M1 | 0.055 | 0.36 |
| Right M1 | -0.053 | 0.37 |
| Bilateral vATL | 0.084 | 0.23 |
| Left vATL | -0.019 | 0.13 |
| Right vATL | 0.044 | 0.13 |
| Bilateral V1 | 0.089 | 0.15 |
| Left V1 | 0.099 | 0.16 |
| Right V1 | -0.068 | 0.16 |

M1: primary motor cortex, vATL: ventral anterior temporal lobe, V1: primary visual cortex.

**Table S40.** Correlations between the wSDM measure and the verb-related information in the NTs, for the PCE group.

| **Seed** | **Pearson’s *r*** | ***p* (FDR-corrected)** |
| --- | --- | --- |
|  |  |  |
| Bilateral M1 | 0.084 | 0.23 |
| Left M1 | -0.094 | 0.21 |
| Right M1 | -0.092 | 0.21 |
| Bilateral vATL | 0.003 | 0.96 |
| Left vATL | 0.018 | 0.99 |
| Right vATL | 0.102 | 0.19 |
| Bilateral V1 | 0.094 | 0.21 |
| Left V1 | -0.081 | 0.24 |
| Right V1 | -0.071 | 0.28 |

M1: primary motor cortex, vATL: ventral anterior temporal lobe, V1: primary visual cortex.

**Table S41.** Correlations between the wSDM measure and the circumstantial information in the NTs, for the PCE group.

| **Seed** | **Pearson’s *r*** | ***p* (FDR-corrected)** |
| --- | --- | --- |
|  |  |  |
| Bilateral M1 | 0.099 | 0.20 |
| Left M1 | 0.115 | 0.17 |
| Right M1 | -0.107 | 0.18 |
| Bilateral vATL | -0.03 | 0.66 |
| Left vATL | 0.116 | 0.17 |
| Right vATL | -0.058 | 0.34 |
| Bilateral V1 | -0.025 | 0.83 |
| Left V1 | 0.104 | 0.19 |
| Right V1 | 0.016 | 0.92 |

M1: primary motor cortex, vATL: ventral anterior temporal lobe, V1: primary visual cortex.

**13. Supplementary data 13. Machine learning analysis without text features**

To test whether linguistic features contributed to subject identification in each group pair, we reran all classification analysis excluding all text-related variables. For the XGBoost classification with the FLE and healthy control groups, we achieved a 70.83% accuracy rate, which proves lower than that obtained upon inclusion of linguistic features (75%). Our feature importance analysis showed higher relevance for the bilateral M1 wSDM feature, followed by ATR FA and the UNC FA. The ROC curve showed an AUC of 0.80, while the confusion matrix scores showed a sensitivity of 75% and a specificity of 66.67% (Figure S1A).

For the XGBoost classification with the PCE and healthy control groups, we achieved a 53.33% accuracy rate, corresponding to classifying by chance. The ROC curve showed an AUC of 0.80, while the confusion matrix scores yielded a sensitivity of 66.67% and a specificity of 40% (Figure S1B).

Lastly, for the XGBoost classification with the FLE and non-FLE groups, we achieved a 63.33% accuracy rate, which, once again, proved lower than that obtained upon inclusion of linguistic features (75%). Our feature importance analysis showed higher relevance for the ATR FA feature, followed by bilateral M1 wSDM and UNC FA. The ROC curve showed an AUC of 0.73, while the confusion matrix scores yielded a sensitivity of 63.33% and a specificity of 60% (Figure S1C).


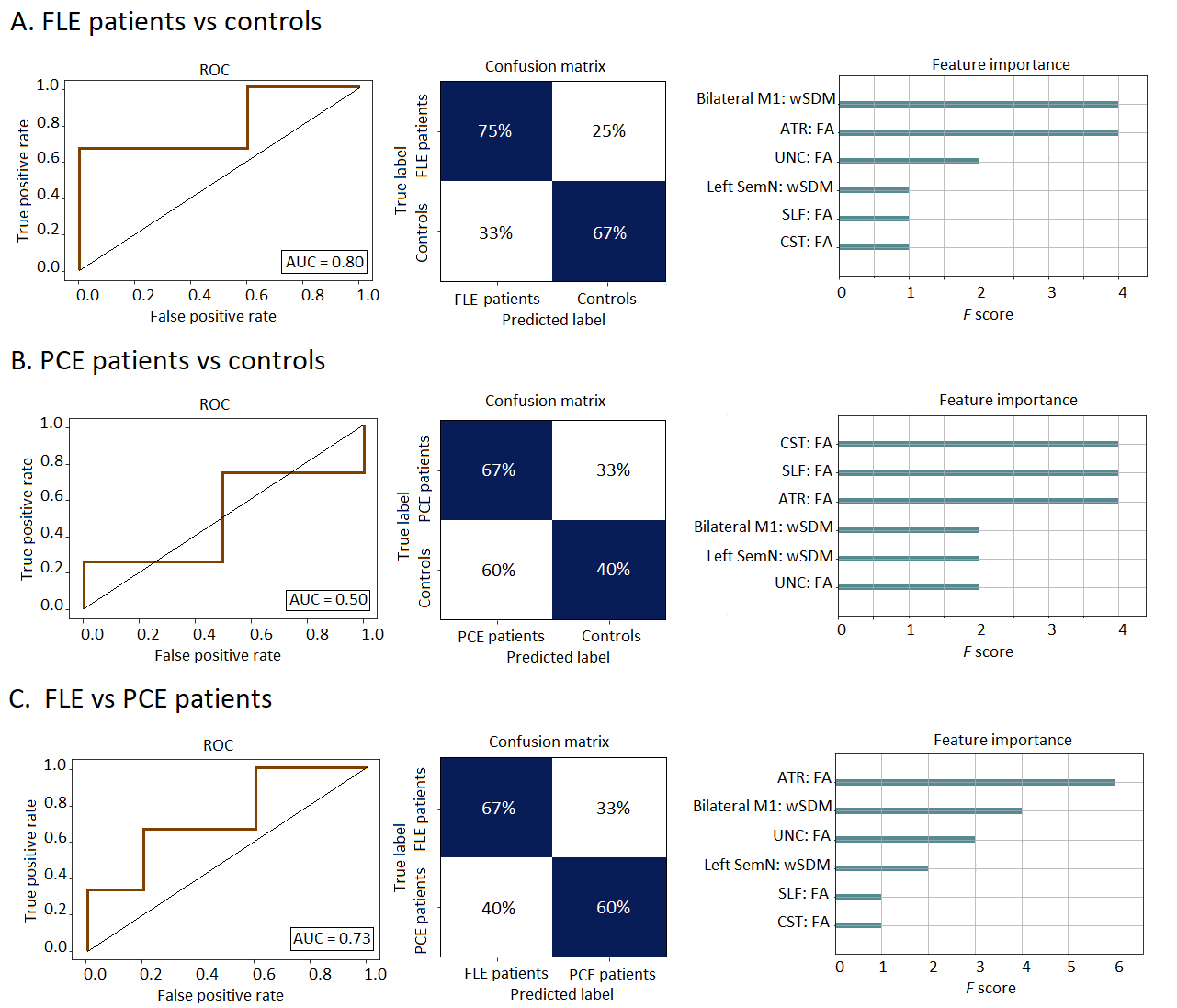


**Figure S1. Supplementary machine learning results**. **(A)** FLE patients vs controls. ROC curve indicating specificity (true positive rate) and sensitivity (false positive rate), while calculating the area under the curve. Confusion matrix for true label and predicted label accuracy details. Feature importance plot of the most relevant features for the classification. Results show a 70.83% accuracy rate, an AUC of 0.80, a sensitivity of 75% and a specificity of 66.67%, with the bilateral M1 wSDM value as the top feature. **(B)** PCE patients vs controls. ROC curve indicating specificity (true positive rate) and sensitivity (false positive rate), while calculating the area under the curve. Confusion matrix for true label and predicted label accuracy details. Feature importance plot of the most relevant features for the classification. The results show near-chance accuracy (53.33%), an AUC of 0.50, a sensitivity of 66.67% and a specificity of 40%. **(C)** PCE vs FLE patients. ROC curve indicating specificity (true positive rate) and sensitivity (false positive rate), while calculating the area under the curve. Confusion matrix for true label and predicted label accuracy details. Feature importance plot of the most relevant features for the classification. The results show a 63.33% accuracy rate, an AUC of 0.73, a sensitivity of 66.67% and a specificity of 60%, with ATR FA as the top feature. ROC: Receiver operating characteristic, AUC: Area under the curve, FLE: Frontal lobe epilepsy, PCE: Posterior cortex epilepsy, ATR: anterior thalamic radiations, SLF: superior longitudinal fasciculus, UNC: uncinate fasciculus, M1: Primary motor cortex, wSDM: weighted Symbolic Dependence Metric.

**14. Supplementary data 14. Machine learning analysis with cognitive features**

The complementary machine learning analyses included: (a) five domain-general measures (subtests of visuospatial, attentional, language, abstraction, and delayed recall from the MoCA) (b) five executive measures (subtests of motor programming, conflicting instructions, inhibitory control, proverb interpretation, and working memory from the IFS), (c) conditions from the naturalistic text tasks, (d) all WM tracts, and (e) all rsFC network features.

The classifier with FLE patients and controls achieved 73.68% accuracy, with 70% sensitivity and 77.78% specificity. The classificatory relevance was highest for the bilateral M1 wSDM feature, followed by verb-related AT scores and the ATR FA, and then by other less relevant features. Classification between PCE patients and healthy controls achieved 55% accuracy, with 60% sensitivity and 50% specificity. The classificatory relevance was highest for the circumstantial AT outcomes, followed by CST tracts, bilateral M1 wSDM, and then by other less relevant features. Finally, classification between FLE and PCE patients achieved 75% accuracy, with 81.82% sensitivity and 66.67% specificity. The classificatory relevance was highest for ATR FA, followed by the bilateral M1 wSDM value and verb-related AT scores, and then by other less relevant features. Se Figure S2 for details.


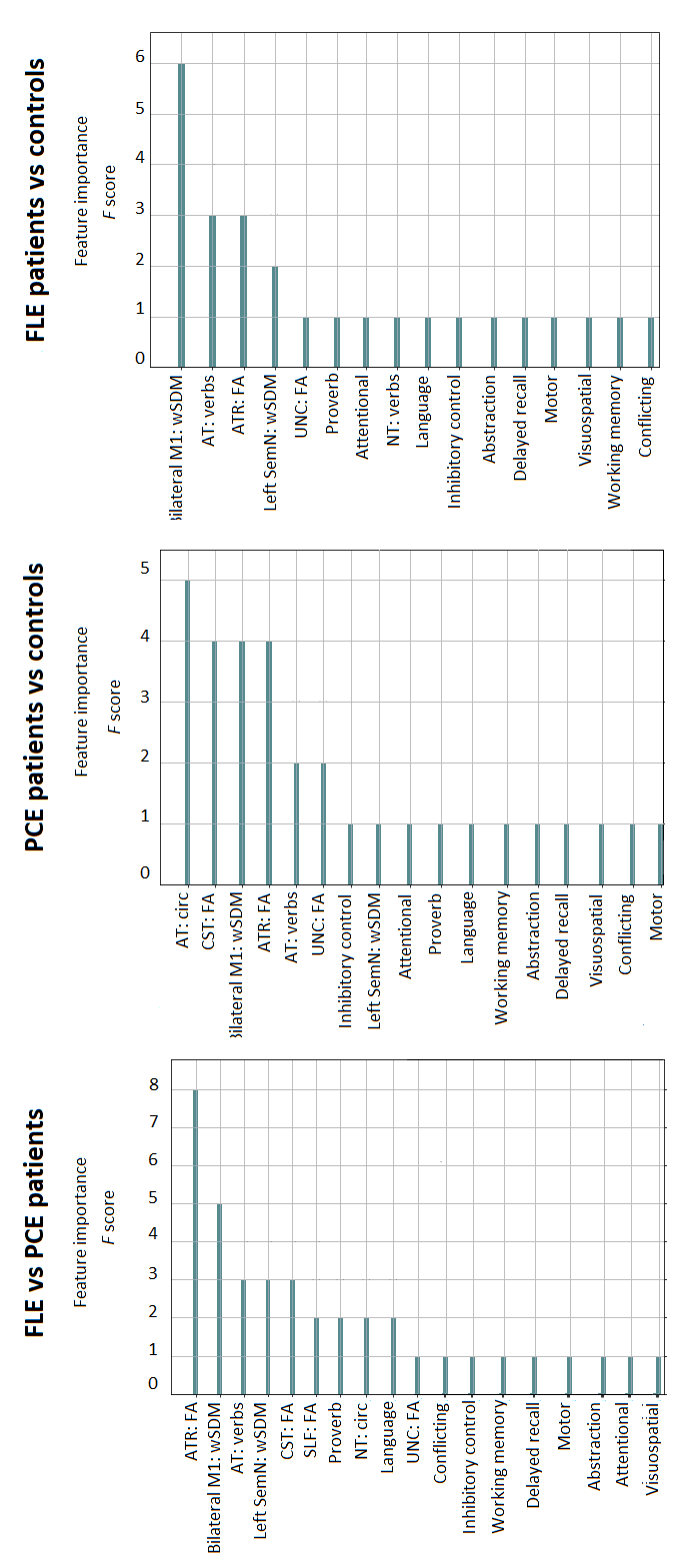


**Figure S2.** Feature importance plot of the most relevant features for the classification including the entire set of features for FLE patients vs. controls, PCE patients vs. controls, and FLE vs. PCE patients.

**References**

1. Faul FE, E. Lang, A.G. Buchner, A. G*Power 3: a flexible statistical power analysis program for the social, behavioral, and biomedical sciences Behav Res Methods. 2007;39:175-191.

2. Garcia AM, Bocanegra Y, Herrera E, Moreno L, Carmona J, Baena A, et al. Parkinson's disease compromises the appraisal of action meanings evoked by naturalistic texts Cortex; a journal devoted to the study of the nervous system and behavior. 2018 Mar;100:111-126.

3. Haselgrove JCM, J.R. . Correction for distortion of echo-planar images used to calculate the apparent diffusion coefficient Magn Reson Med 1996;36.

4. Basser PJM, J. LeBihan, D. Estimation of the effective self-diffusion tensor from the NMR spin echo. J Magn Reson B. 1994;103:247-254.

5. Jenkinson M, Beckmann CF, Behrens TE, Woolrich MW, Smith SM. Fsl NeuroImage. 2012 Aug 15;62:782-790.

6. O'Gorman RL, Bucher HU, Held U, Koller BM, Huppi PS, Hagmann CF, et al. Tract-based spatial statistics to assess the neuroprotective effect of early erythropoietin on white matter development in preterm infants Brain : a journal of neurology. 2015 Feb;138:388-397.

7. Mollink J, Smith SM, Elliott LT, Kleinnijenhuis M, Hiemstra M, Alfaro-Almagro F, et al. The spatial correspondence and genetic influence of interhemispheric connectivity with white matter microstructure Nature neuroscience. 2019 May;22:809-819.

8. Smith SM, Jenkinson, M., Johansen-Berg, H., et al. Tract-based spatial statistics: voxelwise analysis of multi-subject diffusion data NeuroImage. 2006;61:1487–1505.

9. Oishi K, Faria, A., Jiang, H., Li, X., Akhter, K., Zhang, J., Hsu, J. T., Miller, M. I., van Zijl, p. C., Albert, M., Lyketsos, C. G., Woods, R., Toga, A. W., Pike, G. B., Rosa-Neto, p., Evans, A., Mazziotta, J., & Mori, S. Atlas-based whole brain white matter analysis using large deformation diffeomorphic metric mapping: application to normal elderly and Alzheimer's disease participants NeuroImage. 2009;46:486–499.

10. Hua K, Zhang J, Wakana S, Jiang H, Li X, Reich DS, et al. Tract probability maps in stereotaxic spaces: analyses of white matter anatomy and tract-specific quantification NeuroImage. 2008 Jan 1;39:336-347.

11. Chao-Gan Y, Yu-Feng Z. DPARSF: A MATLAB Toolbox for "Pipeline" Data Analysis of Resting-State fMRI Frontiers in systems neuroscience. 2010;4:13.

12. Friston K.J. AJT, Kiebel S.J., Nichols T.E., Penny W.D. Statistical Parametric Mapping: the Analysis of Functional Brain Images. Elsevier/Academic Press. 2007.

13. Song XW, Dong, Z. Y., Long, X. Y., Li, S. F., Zuo, X. N., Zhu, C. Z., He, Y., Yan, C. G., & Zang, Y. F. REST: a toolkit for resting-state functional magnetic resonance imaging data processing PloS one. 2011;6:e25031.

14. Ashburner J, Friston KJ. Nonlinear spatial normalization using basis functions Human brain mapping. 1999;7:254-266. Research Support, Non-U.S. Gov't

15. Vahdat S, Darainy M, Milner TE, Ostry DJ. Functionally specific changes in resting-state sensorimotor networks after motor learning The Journal of neuroscience : the official journal of the Society for Neuroscience. 2011 Nov 23;31:16907-16915.

16. Garcia AM, Moguilner S, Torquati K, Garcia-Marco E, Herrera E, Munoz E, et al. How meaning unfolds in neural time: Embodied reactivations can precede multimodal semantic effects during language processing NeuroImage. 2019 Aug 15;197:439-449.

17. Saiote C, Tacchino A, Brichetto G, Roccatagliata L, Bommarito G, Cordano C, et al. Resting-state functional connectivity and motor imagery brain activation Human brain mapping. 2016 Nov;37:3847-3857.

18. Benjamini Y, & Hochberg, Y. Controlling the False Discovery Rate: A Practical and Powerful Approach to Multiple Testing Journal of the Royal Statistical Society Series B (Methodological). 1995;57:289-300.

19. Poldrack RA, Baker CI, Durnez J, Gorgolewski KJ, Matthews PM, Munafo MR, et al. Scanning the horizon: towards transparent and reproducible neuroimaging research Nature reviews Neuroscience. 2017 Feb;18:115-126.

20. Poldrack RA, Huckins G, Varoquaux G. Establishment of Best Practices for Evidence for Prediction: A Review JAMA psychiatry. 2019 Nov 27.

21. Chen TG, C. Xgboost: a scalable tree boosting system Proceedings of the 22Nd ACM SIGKDD international conference on knowledge discovery and data mining. 2016:785–794.

22. Zeng X, Luo G. Progressive sampling-based Bayesian optimization for efficient and automatic machine learning model selection Health information science and systems. 2017 Dec;5:2.

23. Feurer M. HF. Hyperparameter Optimization. Automated Machine Learning The Springer Series on Challenges in Machine Learning Springer. 2019.

24. Mason LB, J.; Bartlett, p. L.; Frean, Marcus. Boosting Algorithms as Gradient Descent in Function Space. Proceedings of the 12th International Conference on Neural Information Processing Systems. 1999.

25. Behravan H, Hartikainen JM, Tengstrom M, Pylkas K, Winqvist R, Kosma VM, et al. Machine learning identifies interacting genetic variants contributing to breast cancer risk: A case study in Finnish cases and controls Scientific reports. 2018 Sep 3;8:13149.

26. Zheng H, Yuan J, Chen L. Short-Term Load Forecasting Using EMD-LSTM Neural Networks with a Xgboost Algorithm for Feature Importance Evaluation Energies. 2017;10:1168.

27. Torlay L, Perrone-Bertolotti M, Thomas E, Baciu M. Machine learning-XGBoost analysis of language networks to classify patients with epilepsy Brain informatics. 2017 Sep;4:159-169.

28. Xuan p, Sun C, Zhang T, Ye Y, Shen T, Dong Y. Gradient Boosting Decision Tree-Based Method for Predicting Interactions Between Target Genes and Drugs Frontiers in genetics. 2019;10:459.
